# Methanogenesis and Acetogenesis in Hydrogenotrophy with Carbonate Minerals: Dependence on Mineral Surface Area, Biofilm Growth, and Microbial Community

**DOI:** 10.1021/acs.est.4c14291

**Published:** 2025-08-12

**Authors:** Yarong Qi, Sharon Borglin, Langlang Li, Wenming Dong, Markus Bill, Zhao Hao, Céline Pallud, Benjamin Gilbert

**Affiliations:** 1 Department of Environmental Science, Policy, and Management, 1438 University of California, Berkeley, California 94720, United States; 2 Earth and Environmental Sciences Area, 1666 Lawrence Berkeley National Laboratory (LBNL), Berkeley, California 94720, United States; 3 Department of Earth and Planetary Science, 1438 University of California, Berkeley, California 94720, United States

**Keywords:** hydrogen, methanogenesis, acetogenesis, carbonate mineral, calcite, biofilm, Archaea, Bacteria, *Acetobacterium*, *Methanobacterium*

## Abstract

The production, storage, and use of hydrogen are anticipated to grow substantially to achieve energy and climate goals. Consequently, microbial communities in many terrestrial and subsurface Earth environments could be exposed to elevated hydrogen concentrations. Hydrogen stimulates metabolic processes that reduce aqueous chemical species, such as bicarbonate or sulfate, that can exchange with solid mineral phases, but the controls on microbial hydrogenotrophy with mineral sources of electron acceptors are not fully understood. Herein, we applied laboratory experiments and biogeochemical modeling to study the response of a natural microbial community to an elevated partial pressure of hydrogen in the presence of carbonate minerals of varying composition, solubility, and size. Experimental incubations and simulation results showed that hydrogen consumption by microbial communities was initially dominated by sulfate reduction and, subsequently, transitioned to acetogenesis and methanogenesis. The rates of acetogenesis and methanogenesis were not correlated with the solubility of carbonate minerals. Instead, we observed strong linear correlations between the rates and surface area of carbonate minerals. Methane and acetate production slowed down in all incubations after about 2 weeks of incubation, although biogeochemical modeling predicted that the metabolic processes were not thermodynamically limited. Electron microscopy and infrared spectroscopy showed that biofilms with diverse microorganisms grew on the carbonates during this period. The methane δ^13^C value significantly increased, consistent with slower growth at elevated pH. This work highlights that microbial communities form biofilm on carbonate mineral surfaces as a response to hydrogen and that biofilm formation could pose a strong kinetic limitation to hydrogenotrophic metabolism utilizing carbonate minerals.

## Graphical abstract

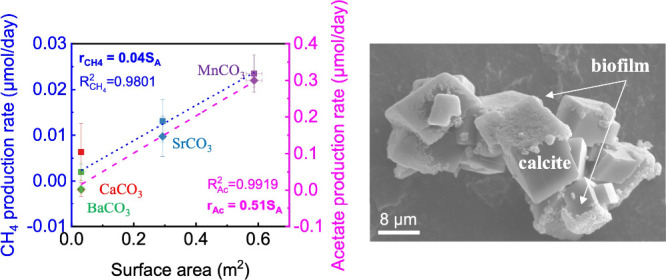

## Introduction

The production, storage, and use of hydrogen from water electrolysis using renewable energy or from natural and stimulated serpentinization are anticipated to grow substantially to achieve energy and climate goals. The growth of anthropogenic hydrogen could cause microbial communities in many terrestrial and subsurface Earth environments to be exposed to elevated hydrogen concentrations. For example, underground hydrogen storage in caverns, natural gas reservoirs, or aquifers would introduce very high local hydrogen concentrations while losses of hydrogen during storage and transport may lead to dispersed elevated hydrogen concentrations in various environments such as groundwater and soils.^1–3^

In anaerobic environments, hydrogenotrophic microorganisms can conserve energy by coupling hydrogen oxidation to the reduction of electron acceptors, such as dissolved carbon dioxide species, sulfate, ferric iron, and nitrate. Anaerobic hydrogenotrophy has been observed in Earth surface systems such as sediments, glacial catchments, soils, ruminant and beetle intestines, and termite mounds where biological processes, including fermentation and nitrogen fixation, generate hydrogen.^4–9^ In previous studies, either pure cultures were used for incubations or metagenomic analysis of environmental samples were applied.^10–13^ Few studies have investigated the geochemical changes under hydrogenotrophic metabolism or the dynamics of microbial community composition after hydrogen exposure.^3,13^

Hydrogenotrophic metabolism has also been reported in studies of subsurface systems influenced by geologic sources of hydrogen, such as serpentinization,^14–16^ and is anticipated to present in underground repositories of radioactive waste.^17^ The anaerobic microbial metabolic processes that are stimulated by hydrogen are well-established (Table S1).^18–24^ However, the geochemical and biological controls of the rate of hydrogen metabolism at elevated partial pressures of hydrogen are not fully known. Often present in trace levels in natural systems, hydrogen can be a limiting substrate for microbial metabolism as determined by thermodynamics,^20^ but few studies have investigated the electron acceptor limitations. Howells et al.^25^ used thermodynamic modeling and microbial community analysis to infer that electron-acceptor limitations could create thermodynamic niches for hydrogenotrophic microorganisms in groundwater supplied with hydrogen from serpentinizing rock. Most laboratory studies have considered hydrogenotrophy with dissolved electron acceptors, while in many settings, solid minerals such as carbonates, sulfates, and ferric iron can represent the largest pool of electron acceptors. Laboratory incubations have shown that carbonate minerals can be the sole carbon source for energy conservation and growth of methanogenic archaea.^13,26,27^ For example, Wormald et al. observed the formation of methane and acetate in incubations of solid carbonate with molecular hydrogen.^27^ The main reactions in the microbial metabolism of hydrogen and carbonate minerals can be described by hydrogenotrophic methanogenesis ([Disp-formula eqR1]) and hydrogenotrophic acetogenesis ([Disp-formula eqR2]). Miller et al.^13^ proposed a conceptual model to explain the features of methane carbon isotope from hydrogenotrophic methanogenesis utilizing carbonate minerals in which mineral dissolution generates dissolved inorganic carbon species, such as bicarbonate (HCO_3_^–^), that are available for microbial uptake and metabolic reactions.

$$4H_{2}+{{\mathrm{HCO}}_{3}}^{-}+H^{+}↔{\mathrm{CH}}_{4}+3H_{2}O$$ Reaction 1

$$4H_{2}+2{{\mathrm{HCO}}_{3}}^{-}+H^{+}↔{\mathrm{CH}}_{3}{\mathrm{COO}}^{-}+4H_{2}O$$ Reaction 2

We explore the conceptual model of Miller et al., asking whether rates of hydrogenotrophy utilizing solid electron acceptors are dependent upon mineral solubility (a thermodynamic concept) or the dissolution rate (a kinetic control). Prior studies of dissimilatory iron reduction demonstrated that mineral solubility can impose a thermodynamic constraint on microbial metabolism by controlling the solution activity of a limiting substrate species.^28^ Here, we test the hypothesis that in the presence of elevated hydrogen, carbonate mineral solubility could impose a thermodynamic constraint on hydrogenotrophy by controlling the solution activity of dissolved carbonate species.

We incubated an anaerobic coastal sediment microbial community with hydrogen (10% at 1 atm) and four metal carbonate minerals. The carbonate minerals differ in their solubility (Table S2) and particle size, which differentiates the effect of aqueous solubility from that of mineral surface area. We investigated the rates of methane and acetate formation in hydrogenotrophy based on geochemical analysis and studied the relationships between the rates and mineral properties, such as surface area and solubility. We further studied the dynamics and compositions of microbial communities in the hydrogenotrophy by 16S rRNA gene sequencing and analyzed the biofilm formed on carbonate mineral surfaces using infrared spectromicroscopy. This work contributes to the understanding of constraints on microbial metabolism and sheds light on the interactions between microbes and minerals in complex environments with elevated levels of hydrogen.

## Materials and Methods

### Chemicals

Synthetic metal carbonate powders of calcium carbonate (calcite, CaCO_3_), manganese carbonate (MnCO_3_), and barium carbonate (BaCO_3_) were purchased from Sigma-Aldrich. Strontium carbonate (SrCO_3_) was obtained from the General Chemical Company (NY). The size and morphology of the minerals were studied by a scanning electron microscopy (SEM) (Zeiss EVO MA-10 SEM) with samples on carbon or copper tape and coated with gold. The specific surface area of carbonates was measured by N_2_ or Kr adsorption (Micromeritics 3Flex) after drying powders with N_2_ flow at 150 °C for 72h. The pure culture of*Acetobacterium wieringae* was purchased from the DSMZ-German Collection of Microorganisms and Cell Cultures and cultured anaerobically with similar procedures used for sediment incubation with carbonate minerals.

### Collection and Incubation of Sediment

Coastal sediment was collected at Meeker Slough, a tidal marsh site (37°54′33.5”N, 122°20′16.4”W) in Richmond, CA, during low tides in July and August of 2022 (Figure S1). Surface sediment with a sampling depth of 0–5 cm was collected into a sterilized plastic bag without air and immediately transferred to laboratory and stored at 4 °C before further operations in anaerobic glovebox. The mineral phase abundance of dry sediment was determined from wide-angle X-ray diffraction (XRD) (Rigaku SmartLab) data using Rietveld refinement in the MAUD software (Figure S2).

The collected fresh sediment was used for stepwise incubation anaerobically with an elevated hydrogen concentration in headspace of serum vials. Hydrogenotrophic metabolism with carbonate minerals using inorganic medium was stimulated by incubating enriched sediment with hyrogen an initial incubation of fresh sediment with hydrogen to enrich hydrogenotrophs. To enrich hydrogenotrophs, first, 1 g of fresh sediment was diluted with 2 g of sterilized degassed DI water in 35 mL sterile vials in sterilized anaerobic glovebox and further incubated with 10%–20% hydrogen in Ar (filtered with 0.1 μm sterile filter) in vial headspace at 30 °C for a week in the dark under constant orbital shaking (120 rpm). Second, the enriched sediment fluid was further incubated at 30 °C with 10% hydrogen in Ar or He in headspace and with inorganic medium^27^ (Tables S3 and S4) (a volume ratio of enriched fluid to medium of 1:30) amended with 20 g/L carbonate minerals of different solubilities (Table S2) to stimulate hydrogenotrophic metabolic reactions, which are favorable under standard conditions and standard physiological conditions (Text S1, Tables S5, S6, and S7). Control experiments are incubation of enriched sediment with hydrogen without carbonate minerals in inorganic medium, incubation of carbonates in inorganic medium with hydrogen but without enriched sediment, and incubation of enriched sediment with hydrogen and 10% CO_2_ or 10–50 mM bicarbonate in inorganic medium. Additionally, further incubation at 30 °C of the enriched sediment in the inorganic medium (a volume ratio of enriched fluid to medium of 1:300) with 20 g/L calcite to develop biofilm for SEM imaging or with 20 g/L silica microspheres as control experiment.

### Geochemical Analyses

During the stepwise anaerobic incubation of sediments, the gas phase in headspace of vials was sampled with two-day intervals for a month and analyzed by a gas chromatograph (GC-8AIT, Shimadzu, detection limit of 0.1% for hydrogen) and the concentrations of hydrogen and methane in the gas phase were further calculated. Acetate, sulfate, nitrate, formate, cations (e.g., Ca^2+^, Sr^2+^, Mn^2+^, and Ba^2+^), and total organic and inorganic carbon in fluid samples were measured by an Ion Chromatograph (IC, Metrohm 881), Agilent 8900 QQQ Inductively Coupled Plasma Mass Spectrometry (ICP-MS), and Total Organic Carbon Analyzer (TOC-L, Shimadzu Corporation), respectively. The carbon stable isotope composition of methane was analyzed by a Thermo Scientific GC Trace Gas Ultra interfaced to a combustion system (for carbon, 1030 °C, Ni/Cu/Pt catalyzed) or pyrolysis furnace (for hydrogen, 1450 °C) connected to a Thermo Scientific Delta V Plus Mass Spectrometer (IRMS).

Initial rates of hydrogenotrophic methanogenesis and acetogenesis were calculated once the products of these metabolic processes became detectable within 7–14 days of incubation based on these processes (eq 1).

$$r_{i}=\frac{\Delta n}{\Delta t}$$ 1

where *r*_*i*_ in μmol/day is the initial rate of a metabolism *i*, and *i* represents either methanogenesis or acetogenesis in this work. The symbol Δ*n* in μmoles is the change in the amount of a reaction product that occurred over a change of time Δ*t* in days.

### Biogeochemical Modeling

Biogeochemical modeling was applied to predict the main metabolic processes in hydrogenotrophy with carbonate minerals. The rates of microbial growth via hydrogenotrophic metabolic processes coupled to geochemical and mineralogical changes were calculated using the Geochemist’s Workbench (GWB) using the default thermodynamic database. All of the rate equations and their parameters and input scripts are given in Text S2. The microbial metabolism rate laws predict the rate of biomass generation as a function of dissolved electron donor and acceptor concentrations. The GWB code was chosen because the built-in metabolic rate laws combined a generalized form of the Monod expression with the thermodynamic factor introduced by Jin and Bethke.^29,30^ The thermodynamic factor accounts for the free energy that is required for each model microorganism to synthesize a certain number of adenosine triphosphate molecules (ATP) *per* metabolic cycle. Starting parameters for the rate laws for sulfate reduction and methanogenesis were obtained from the literature.^30^ Starting parameters for acetogenesis were not found in the literature and were set to the same values as methanogenesis.

The built-in GWB model framework cannot simulate dynamic gas exchange between an aqueous phase and a finite-volume gas reservoir. Thus, in order to compare simulation with experiment, the time dependence of the gas phase hydrogen and methane concentrations was calculated from the amount of gas transfer via a kinetic term. The GWB input scripts for microbial growth with hydrogen and calcite or rhodochrosite are given in the Supporting Information, and all rate law parameters are given in Tables S8 and S9.

The modeling used here is intermediate in complexity compared with prior studies. Hemme and van Berk^31^ used PHREEQC to predict the subsurface utilization of hydrogen by microbial sulfate reduction and methanogenesis using the Monod equation. However, the metabolism model cannot describe how the electron acceptor activity may impose a thermodynamic limit on hydrogenotrophy. Gropp et al.^32^ developed a detailed kinetic model for all steps of acetoclastic methanogenesis by *Methanothermobacter thermoautotrophicus* considering the free energy changes of individual reaction steps and effects of the departure of each step from equilibrium on the hydrogen isotope ratio of the intermediates and the methane product. However, this kinetic model is not coupled to solution geochemistry, including mineral dissolution, and is not suited to understanding hydrogenotrophy involving mineral sources of electron acceptors.

### Microbial Community Analysis

Aliquots of 0.5–1 mL of the incubated fluids were collected at a few time points during 21 days of incubation before centrifugation at 10,000*g* for 5 min to pellet cells for total DNA extraction. The collected pellets and 500 mg of raw sediment were subject to total genomic DNA extraction with a commercial kit Qiagen DNEasy Powersoil Pro Kit (Qiagen, Manchester, UK). Samples were incubated in triplicate, and the DNA was combined for sequencing. The DNA was quantified using a Qubit 3 fluorometer (Invitrogen).

A primer pair 340*F*/806R was used to amplify the V3–V4 region of 16S rRNA gene of archaeal and bacterial DNA.^33^ Details are given in Text S3 in the Supporting Information. The 16S rRNA gene-based amplicon sequencing was performed on an Illumina Miseq platform with 2 × 300 bp paired-end sequencing mode at QB3 Genomics, UC Berkeley. The community composition and diversity were analyzed on QIIME2 (details in Text S4 and S5 in the Supporting Information).

### Biofilm Characterization

Cells and biofilms on carbonate mineral surfaces were imaged by SEM. A droplet of incubated suspension was filtered on a 0.2 μm filter membrane before dehydration with a series of diluted ethanol solutions with 20%, 40%, 60%, 80%, and 100% (volume ratio) of ethanol. The dehydrated sample was coated with a layer of gold to reduce the level of charging during SEM imaging.

The biochemical profile of the biofilm samples was analyzed with an Optical Photothermal Infrared (O-PTIR) microscope (mIRage-LS, Photothermal Spectroscopy Corp.) at the Infrared Beamlines 1.4 of the Advanced Light Source, LBNL. This microscope exploits the photothermal effect induced by modulated infrared radiation, which is subsequently detected by a visible laser beam. A group of cascade lasers were employed to cover the mid-IR frequency range of 800 to 1800 cm^–1^. The diffraction-limited submicron spatial resolution, determined by the 532 nm wavelength of the visible probe laser, makes it compatible with our target microbes.^34^ This also eliminates the background signal from the carbonate group at ∼1450 cm^–1^, due to the attenuation effect of the biofilms. A generic assignment of IR absorbance spectra to biological macromolecules includes (a) lipids by ester peaks at 1800–1700 cm^–1^, (b) proteins or peptides, with amide I and amide II bands at 1700–1500 cm^–1^, (c) nucleic acids with mixed phosphodiester bands and amide II and amide III at 1500–1200 cm^–1^, (d) polysaccharides marked by cell-wall carbohydrates at 1200–900 cm^–1^, and (e) a fingerprint region with spectral features not yet assigned to known cellular components, at 900–800 cm^–1^.^35–38^

## Results and Discussion

### Rates of Hydrogenotrophic Methanogenesis and Acetogenesis

Hydrogenotrophic methanogenesis and acetogenesis were stimulated by incubation of enriched sediment with carbonate minerals under an elevated hydrogen concentration. In a control experiment, sediment in inorganic medium that was incubated with hydrogen but without the addition of any form of CO_2_ consumed negligible hydrogen and did not produce acetate or methane (Figure S3A). Control experiments with the incubation of carbonate minerals in inorganic medium with hydrogen but without the sediment do not consume hydrogen. Incubation of the sediment with hydrogen and sodium bicarbonate in inorganic medium generated micromolar quantities of methane but did not produce acetate above the initial concentration (Figure S3B). In contrast, sediment incubations with hydrogen and the different carbonate minerals in inorganic medium produced both methane and acetate (Figure S4). The produced methane and acetate can be attributed to microbial metabolism utilizing the dissolved carbon species provided by the dissolution of carbonate minerals, as indicated by the increases in inorganic carbon, pH, and cations (Figures S5 and S6) in incubation fluids.

The geochemical changes are clearly illustrated for sediments incubated with calcite (Figure 1A). Hydrogen initially causes the loss of sulfate from residual sediment porewater, attributed to microbial sulfate reduction, accompanied by the loss of iron, attributed to iron sulfide precipitation. Methane was detected after approximately 10 days, when the acetate concentration also rose above the initial level, consistent with hydrogenotrophic methanogenesis and acetogenesis. The solution pH increased around 0.1 pH unit, consistent with the stoichiometry of these metabolic reactions that consume protons. In the calcite and all other carbonate mineral incubations, methane production slowed down significantly, and acetate production either plateaued or declined after around 20 days.

**Figure 1:**
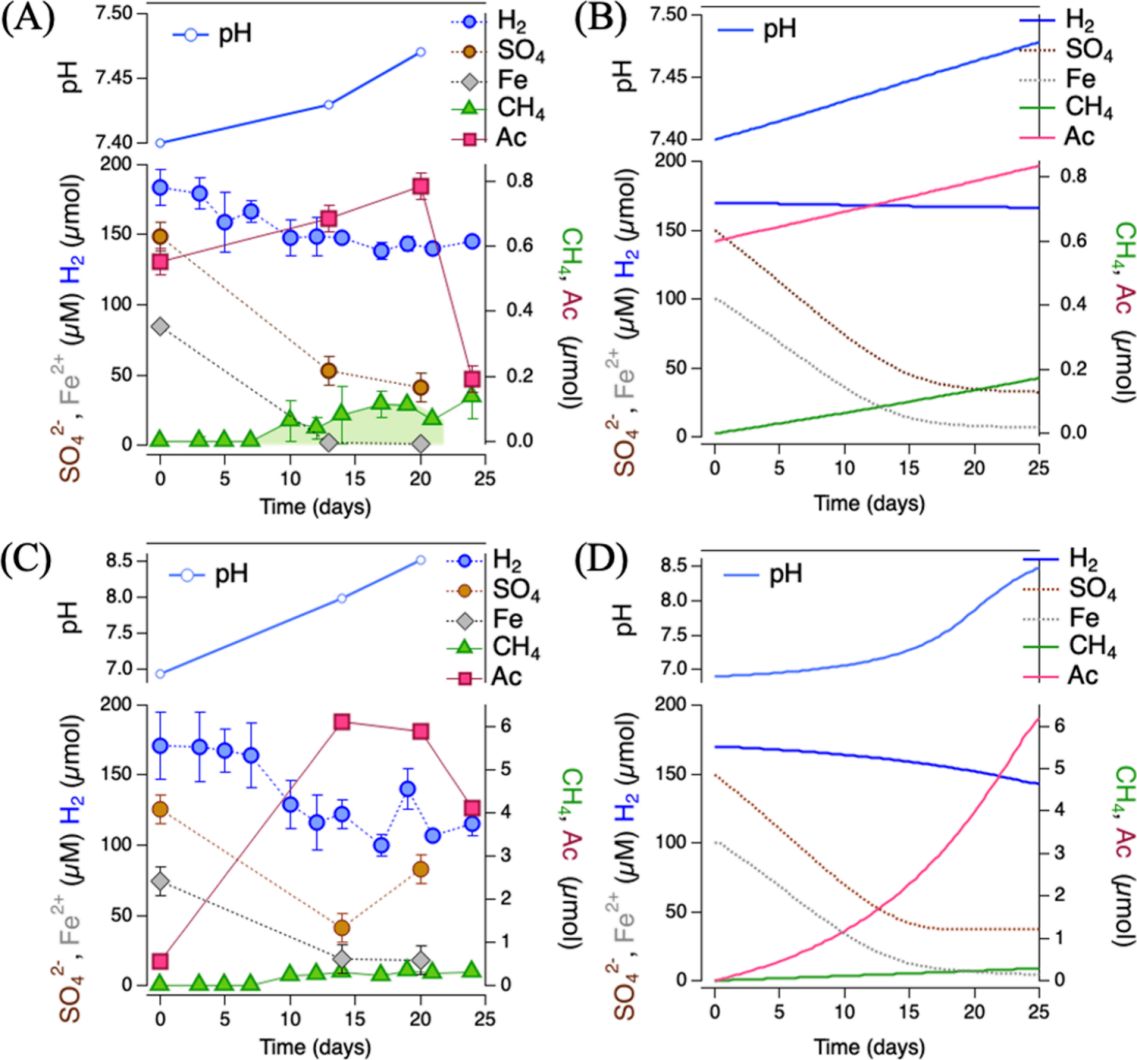
Temporal evolution of pH (light blue), and concentrations of hydrogen (H_2_, dark blue), sulfate (SO_4_, orange), iron (total Fe in aqueous phase, gray), methane (CH_4_, green), and acetate (Ac, pink) during anaerobic incubation of 1% (g/g) sediments with 20 g/L of the carbonate minerals CaCO_3_ (top panels) and MnCO_3_ (bottom panels) in an inorganic medium under 10% (v/v) hydrogen at 30 °C. (A, C) Observed concentrations, (B, D) modeled concentrations.

The sediment incubations with hydrogen and other carbonate minerals, such as manganese carbonate (Figure 1B) and strontium and barium carbonate (Figure S4) stimulated the same metabolic and geochemical processes but at different rates. The rates of methanogenesis and acetogenesis within 20 days of incubations, however, were not significantly correlated with thermodynamic parameters such as carbonate mineral solubility (Figure S7 and Table S2). Indeed, the least soluble mineral, MnCO_3_, showed the fastest rates of methane, 0.02 μmol/day, and acetate, 0.30 μmol/day, production. It is unlikely that Mn stimulated extra growth since adequate Mn was present in all trace medium and because Mn and P levels dropped significantly in this experiment, likely due to the precipitation of manganese phosphate. In addition, the rates of methanogenesis and acetogenesis did not match the trend in the concentration of bicarbonate ions in solution (Figure S6), nor the rates follow the trends in free energy of the metabolic reactions when calculated either using a conventional reaction stoichiometry involving only dissolved species or considering a reaction including solid carbonate mineral dissolution (Figure S8, Tables S5–S7).

In contrast, we discovered statistically significant linear relationships between the initial rates of methanogenesis, *r*_CH_4__, acetogenesis, *r*_Ac_, and the mineral surface area, *S*_A_, (Figure 2). The relationships were quantified by fits of the form *r*_i_ = *k*_i_ *S*_A_, where *i* is either CH_4_ or acetate (Ac). The fitted apparent rate constants for hydrogenotrophic methanogenesis and acetogenesis at 30 °C are *k*_CH_4__ = 0.04 ± 0.00 μmol/m^2^/day and *k*_Ac_ = 0.51 ± 0.03 μmol/m^2^/day, respectively. Thus, the rate of acetogenesis was close to 10 times the rate of methanogenesis *per* unit area of carbonate mineral.

**Figure 2:**
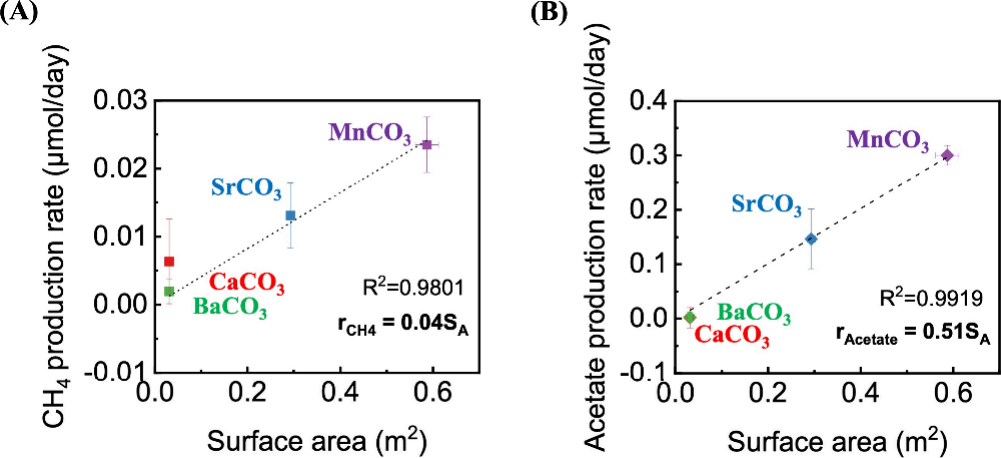
Rates of (A) hydrogenotrophic methanogenesis and (B) acetogenesis correlated to surface area of the carbonate minerals MnCO_3_ (purple), SrCO_3_ (blue), CaCO_3_ (red), and MnCO_3_ (green). Error bars represent the standard deviation. The dotted lines indicate linear fitting.

The rates of metabolic reactions could influence the isotopic characteristics of the products. We also analyzed the methane carbon isotope composition of hydrogenotrophic methanogenesis from 7 to 21 days of sediment incubation with carbonate minerals (Figure S9). At the seventh day of incubation, the δ^13^C values of methane, δ^13^C_CH4_, were −68 and −80 ‰ for incubations with CaCO_3_ and MnCO_3_, respectively, which are consistent with ^13^C depleted methane from microbial processes. Then, δ^13^C_CH4_ values increased to over −27 and −34 ‰ for CaCO_3_ and MnCO_3_, respectively, within 21 days of incubation. The result of high values of δ^13^C_CH4_ is consistent with high δ^13^C_CH4_ from methanogenesis under alkaline conditions as reported by Miller et al.,^13^ challenging the consensus of high carbon isotope (δ^13^C_CH4_ > −40‰) of abiotic methane. This finding shows the time dependence of δ^13^C_CH4_ in hydrogenotrophic methanogenesis.

### Microbial Community Composition

To fill the gap of investigation on the dynamics of microbial community evolution upon environmental interventions, we studied the abundance and diversity of microbial communities in the hydrogenotrophy with carbonate minerals by time series 16S rRNA gene sequencing and analysis. Sediment incubation with hydrogen, with either soluble or mineral phase carbonates, stimulated the growth of hydrogenotrophs, as indicated by increasing trends of total DNA and total DOC (Figure S10) and caused significant changes in microbial community diversity and composition (Figure 3 and Figures S11–S16). Hydrogen stimulation caused a significant loss in diversity (Figure S11), though with a lesser diversity loss for incubations with carbonate minerals than with bicarbonate. Changes in the relative abundance of microbial community based on 16S rRNA gene analysis suggest a shift from bacteria- to archaea-dominated communities (Figure S12), though it could be biased by the use of certain primer pairs. The microbial community in initial sediment showed a highest diversity with over 99.98% of microorganisms at a genus level get lost in hydrogen stimulated sediments. Hydrogen incubations stimulate microorganisms in both bacterial groups *Acetobacterium*, *Erysipelothrix*, *Desulfomicrobium*, and *Thauera*, and archaeal groups *Methanobacterium*, *Methanococcoides*, *Methanolobus*, and *Methanosarcina* at a genus level (Figures S12–S14). The relative abundance of a typical hydrogenotrophic acetogenic genus, *Acetobacterium*, was significantly increased after sediment incubation with hydrogen, and so was the relative abundance of a sulfate-reducing genus, *Desulfomicrobium* (Figure S15). Hydrogen incubation also significantly selected a typical hydrogenotrophic methanogenic genus, *Methanobacterium*, with stepwise incubations, resulting in the *Methanobacterium*-dominated microbial communities (Figure S15). However, the resultant abundance of microorganisms in a microbial community is subject to a few factors, such as total DNA extraction practice and primer selection; thus, a quantitative comparison between functional microbial groups is not applicable. Principal component analysis (PCA) performed based on sequencing reads showed distinct groupings of communities from raw sediment, hydrogen stimulated sediment, and carbonate incubations, further distinguishing sodium bicarbonate incubated sediment from mineral incubated sediment (Figure S16).

**Figure 3:**
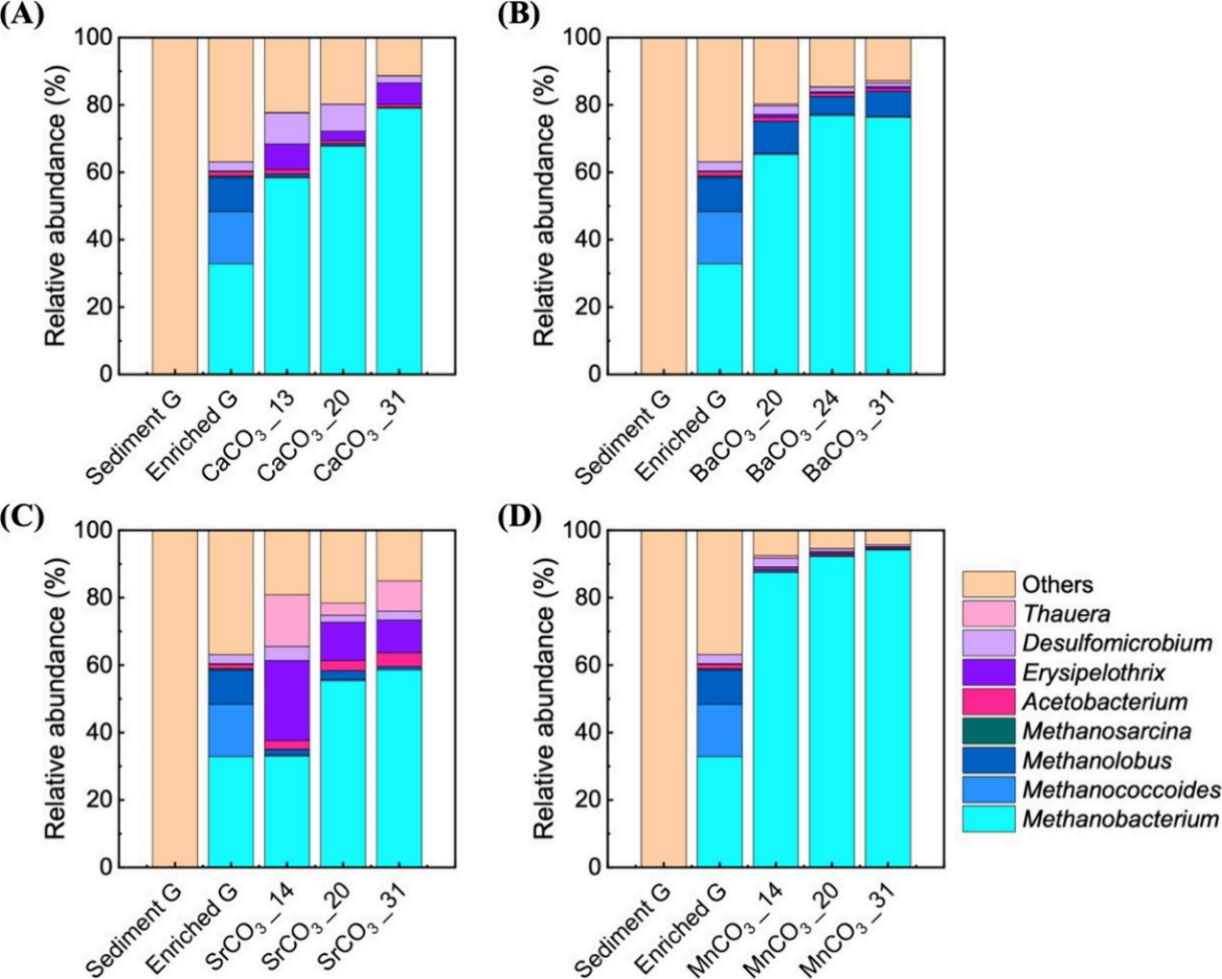
Changes in the relative abundance of prokaryotes at a genus level in the initial sediment, hydrogen-enriched sediment, and carbonate incubations with (A) CaCO_3_, (B) BaCO_3_, (C) SrCO_3_, and (D) MnCO_3_. In all of the incubations with carbonate minerals, the four most abundant archaeal genera and the four most abundant bacterial genera were the same. The plots show the relative abundances, as determined by the number of sequence reads, for all samples. Over 99.98% of the initial organisms from the initial sediment were lost during incubations. The DNA extracts from the replicates were pooled together for sequencing.

### Biogeochemical Modeling

Biogeochemical modeling that included sulfate-reducers, acetogens, and methanogens was performed for incubations with CaCO_3_ and MnCO_3_ (Figure 1B,D). For each simulation, the rates of the metabolic pathways were varied by hand to match trends in reactants (i.e., H_2(*g*)_ and SO_4_^2–^_(*aq*))_ and products (i.e., acetate_(*aq*)_ and CH_4(*g*)_) observed within the first 20 days. Fair agreement was achieved by starting with literature metabolic rate law parameters for each reaction and adjusting, as necessary, one kinetic parameter, *k*_+_, the microbial reaction rate constant, and one thermodynamic parameter, *n*_ATP_, the number of moles of ATP produced per reaction. The *n*_ATP_ value was set equal for both acetogenesis and methanogenesis simulations since the microbial composition was similar at the genus level. In addition, the mineral dissolution rate constant for rhodochrosite was increased relative to that for calcite to account for the higher surface area. All parameters are given in Tables S8 and S9.

The simulation underestimated hydrogen consumption compared to the experiments, indicating, as discussed below, the occurrence of geochemical or microbial processes that were not included in the model. Nevertheless, the simulations accurately account for the stoichiometric changes in carbonate species required for acetate and methane production and reveal the complex coupling between the metabolic consumption of bicarbonate and changes in pH and mineral stability. For example, simulations of the calcite incubations predict that more calcite precipitates due to the pH increase despite the consumption of bicarbonate for hydrogenotrophy, while rhodochrosite is dissolved despite the more substantive increase in pH.

In all incubations, sulfate reduction occurred promptly, aided by the higher available free energy for this pathway compared with acetogenesis and methanogenesis but did not proceed to completion. The biogeochemical modeling suggest that sulfate reduction may have ceased due to the loss of thermodynamic drive once ATP production was considered, despite the presence of excess hydrogen and residual sulfate (Figure 1A,C and Figure S17). Acetogenesis may have slowed down due to the loss of thermodynamic drive in the MnCO_3_ incubation that generated the most acetate and the highest pH (Figure 1C and Figure S18). However, acetate production also ceased in the calcite experiment that modeling suggests is not likely due to metabolic thermodynamic constraint based on bulk fluid composition. Methanogenesis was always thermodynamically favorable.

### Biofilm Development on Carbonate Mineral Surfaces

SEM imaging of incubated samples showed complex associations of biological matter with solids including carbonate grains, clay minerals, and likely dead plant material. In these samples, it was difficult to identify individual microbial cells and carbonate mineral particles. More interpretable SEM images were obtained for a second series of incubations performed at a 10-times-lower ratio of sediment to carbonate mineral. SEM imaging of these samples clearly revealed the attachment of microbial cells to carbonate surfaces (Figure S18) and the growth of apparent biofilms that increasingly coated the carbonate mineral surfaces over time (Figure 4A). The SEM imaging showed both rod-like cells, possible acetogens,^39^ and spheroidal cells and cell clusters, possibly methanogens,^10,40,41^ though those microbes can be diverse in morphology and metabolism.^42,43^ The presence of methanogens in biofilm was suggested by the fluorescent imaging of biofilm on calcite surfaces with F_420_ autofluorescence as the indicator of methanogens (Figure S19). Acetogen-calcite association was also observed from *Acetobacterium wieringae* pure culture incubation with calcite (Figure S20).

**Figure 4:**
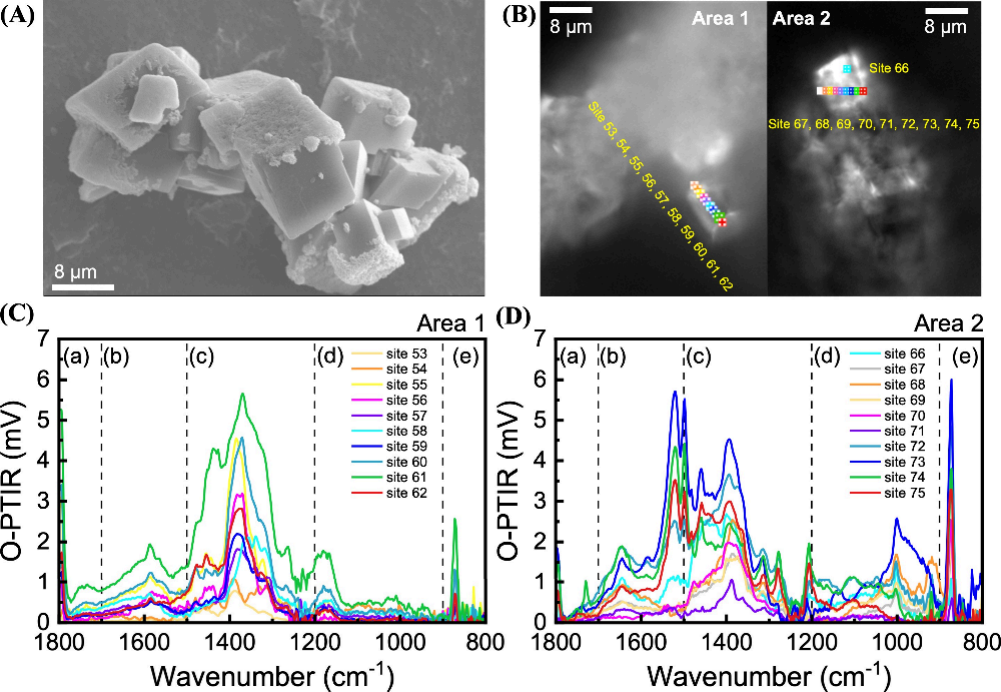
(A) SEM image and (B) visible images with marked locations of optical photothermal infrared (O-PTIR) spectroscopy characterization of biofilm on Area 1 and Area 2 of surfaces of calcite from incubation of 1,000-fold diluted enriched sediment with calcite in inorganic medium, and (C) and (D) IR absorbance spectra assigned to biological macromolecules including (a) lipids, (b) proteins or peptides, (c) nucleic acids, (d) polysaccharides, and (e) unknown fingerprint components.

O-PTIR microspectroscopy provides further evidence that the calcite surfaces were coated with organic polymers including (a) lipids, (b) proteins or peptides, (c) nucleic acids, and (d) polysaccharides, consistent with macromolecules originated from cells and biofilms (Figure 4B and Figure S21). Two distinguishable biochemical profiles were associated with two regions on calcite surfaces labeled as Area 1 and Area 2, respectively, indicating different stages of biofilm growth. In Area 1, the IR signatures revealed a higher content of lipids, nucleic acids, and proteins (Figure 4C), likely associated with higher counts of microbial cells. In contrast, Area 2 exhibited a region rich in polysaccharides, indicating the presence of extracellular polysaccharides and cell wall carbohydrates in the biofilm (Figure 4D).

### Hydrogenotrophy with Carbonate Minerals Coupled to Biofilm Growth

The incubation of an anaerobic sea marsh sediment with hydrogen and carbonate minerals caused biogeochemical changes, including methanogenesis, acetogenesis, and sulfate reduction, that were observed in prior studies.^13,27^ Hydrogen stimulated several hydrogenotrophic metabolic processes, the community structure became significantly less diverse, and the pH rose. This work tested the hypothesis that hydrogenotrophy using carbonate electron acceptors is controlled by the mineral solubility. However, we found that the rates of hydrogenotrophic methanogenesis and acetogenesis did not follow mineral thermodynamic trends but were linearly correlated with mineral surface area. This finding contrasts with the prior observation^30^ that the rate of iron (oxyhydr)­oxide reduction by the iron reducing bacterium *Shewanella putrefaciens* with lactate as the electron donor correlated positively with the solubility of the Fe­(III) oxyhydroxides.

The biofilm containing methanogens and possibly acetogens formed on the carbonate surfaces. Biofilm formation on carbonate minerals has been previously observed in hydrogen rich environment, such as archaeal biofilms in the marine hydrothermal setting dominated by Lost City *Methanosarcinales*.^12^ Recently, the addition of solid inert particles to pure cultures of methanogens increased the rate of methane production.^44^ In our study, however, the addition of silica microspheres did not lead to significant cell attachment. Therefore, our work presents the unique roles of carbonate minerals as electron acceptor, carbon source, and support for biofilm development.

The trends in methanogenesis and acetogenesis in the incubations are likely associated with the formation of biofilm on the surfaces of the carbonate minerals (Figure 5). Both hydrogen-stimulated acetogenesis and methanogenesis ceased after about 3 weeks of incubation despite the availability of metabolic substrates and necessary nutrients. Bulk fluid geochemical factors do not appear to explain this observation, as the bulk aqueous fluid pH remained within a suitable range of 7.5–8.5 that was expected for microbial viability. Timing suggests that these metabolic processes dramatically slowed down when the carbonate mineral surfaces are fully covered by biofilm. When the microorganisms consume bicarbonate from the dissolution of the carbonate mineral surface, a much larger increase in biofilm pH could occur that ultimately make the local environment nonviable for cellular physiology. In addition, the development of biofilm on carbonate mineral surfaces inhibits the carbonate mineral dissolution and limits the dissolved bicarbonate that can be utilized by microbes in the outer layers of the biofilm that, again, could lead to the inhibition of the hydrogenotrophy. The lowered dissolved bicarbonate may also contribute to the increased δ^13^C_CH4_, which aligns well with the high δ^13^C values of methane from both experimental and modeling results by Miller et al.^13^

**Figure 5:**
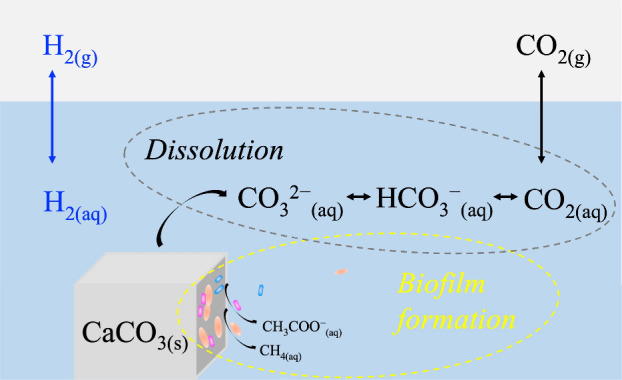
Conceptual model of coupled surface processes of carbonate mineral dissolution and biofilm formation.

However, the hydrogenotrophic methanogenesis (0.005 μmol/day for calcite, which is 2.20 μmol/m^2^/day and hence 2.54 × 10^–15^ mol/cm^2^/s) is relatively slow, which corresponds to the lowest dissolution rate of calcite reported for calcite dissolution in oceanic environments (in an order of 10^–14.6^ mol/cm^2^/s),^45^ and is lower than observed calcite dissolution rate (12.2 μmol/m^2^/day, which is 1.42 × 10^–14^ mol/cm^2^/s, 10^–13.8^ mol/cm^2^/s) after 48 h of dissolution in abiotic experiments at 30 °C in this work, which is close to the lower limit of calcite dissolution rates (10^–13.8^ mol/cm^2^/s) reported by others.^46^ Finally, the hydrogenotrophic acetogenesis is much faster, about 10 times the rate of methanogenesis, the interpretation of which requires further investigation in future work.

The simulations that include acetogenesis, methanogenesis, and sulfate reduction do not account for all the hydrogen that was consumed in the experiment, indicating a role for other metabolic pathways, such as dissimilatory iron reduction. Although crystalline ferric iron (oxyhydr)­oxides were not observed in the XRD data, a small increase in the relative abundance of the genus *Ferrimonas* was observed in all carbonate mineral incubations. At least one species of *Ferrimonas* has iron reduction capability^47^ and while the taxa abundance was always less than 1%, iron reduction may have contributed to hydrogen consumption. Formate generation could not be the reason for extra hydrogen consumption because formate was below detection limit in our incubations though it was reported as a product of hydrogenotrophy in laboratory incubations of anaerobic rock and fluid from a subsurface aquifer.^48^ Formate is an intermediate and potential product of the acetogenesis pathway and is correlated with the abundance of *Acidobacteria*. However, the relative abundance of *Acidobacteriota* in this study remained at 0.01% or less. Interestingly, there are dominantly methanogens in Na_2_CO_3_ incubations while more acetate than methane in carbonate mineral incubations. Future work will be needed to understand how carbonate mineral surfaces regulate activities of acetogens and methanogens.

## Implications

With the growing production and use of hydrogen in natural and engineered environments, it is vital to broaden our understanding of the pathways of microbial communities responding to the increase in environmental hydrogen. In many natural environments, carbonate minerals are the major reservoir of carbon dioxide that can be utilized by hydrogenotrophic organisms. Our experimental and modeling results reveal that bacterial and archaeal microorganisms with the capacity for hydrogenotrophy can initiate acetogenesis and methanogenesis simultaneously and form biofilms on carbonate mineral surfaces. This study was designed to test a mechanistic hypothesis and provides a quantitative relationship with a very high statistical significance. Specifically, we found that the rates of acetogenesis and methanogenesis were linearly dependent on the surface area of carbonate minerals and not determined by thermodynamic constraints (the solubility of these minerals) through experiments and biogeochemical modeling. We speculate that the hydrogenotrophy with carbonate minerals was kinetically limited initially due to the slow carbonate dissolution and later because of the biofilm growth and coverage on the carbonate mineral surfaces, which could potentially limit the availability of dissolved bicarbonate for the hydrogenotrophy. These findings strongly indicate the important roles of surface processes, including mineral dissolution and biofilm formation, in the stimulated metabolism of natural microbial communities in any environments. This work is of special importance for future studies on the evaluation of the interactions between microbes and minerals at the water-mineral interface in complex natural and engineered environments in the context of natural and stimulated serpentinization and subsurface hydrogen storage.

## Supplementary Material



## Notes

Text S1: Calculation of Gibbs free energy change of reaction; Text S2: Biogeochemical modeling with the Geochemist’s Workbench (GWB); Text S3: Polymerase Chain Reaction (PCR) of 16S rRNA gene; Text S4: Microbial community analysis on QIIME2; Text S5: Code for microbial community analysis on QIIME2; Table S1: Microbial metabolic processes stimulated by hydrogen; Table S2: The solubility of carbonate minerals at 25 °C and the measured surface area; Table S3: Inorganic medium composition; Table S4: Trace element solution composition; Table S5: Gibbs free energy changes of methane and acetate production by hydrogen oxidation coupled to bicarbonate reduction under standard condition and physiological standard condition; Table S6: Gibbs free energy changes of methane production by hydrogen oxidation coupled to the reduction of carbonate mineral under standard condition and physiological standard condition; Table S7: Gibbs free energy changes of acetate production by of hydrogen oxidation coupled to the reduction of carbonate mineral under standard condition and physiological standard condition; Table S8: The rate law parameters for GWB simulation of microbial growth with hydrogen and calcite (CaCO_3_) or rhodochrosite (MnCO_3_); Table S9: The rate law parameters for GWB simulation of carbonate mineral dissolution of calcite (CaCO_3_) or rhodochrosite (MnCO_3_); Figure S1: Location and photographs of the coastal sediment collection site; Figure S2: Mineral phase abundance in anaerobic sediment; Figure S3: Time dependence of hydrogen, methane, and acetate concentrations in control experiments; Figure S4: Time dependence of the generation of gas-phase hydrogen and methane and aqueous acetate during the incubation of enriched sediment with 10% hydrogen and 20g/L carbonate minerals; Figure S5: Time dependence of calculated concentrations of bicarbonate (HCO_3_^–^), measured concentrations of inorganic carbon (IC), and measured pH during the incubation of enriched sediment with hydrogen and the carbonate minerals; Figure S6: The dynamics of carbonate mineral derived cation in micromoles and cation concentrations in incubations with carbonate minerals; Figure S7: Initial rates and normalized initial rates of hydrogenotrophic methanogenesis and acetogenesis correlated to the carbonate mineral solubility (25 °C, Table S2) and the inverse of solubility; Figure S8: The dynamics of free energy changes, Δ*G*_r_, of hydrogenotrophic methanogenesis and acetogenesis calculated based on consumption of bicarbonate ions for methane production and acetate production (Table S5), and consumption of carbonate minerals for methane production and acetate production (Tables S6–S7); Figure S9: Measured time-dependence of carbon isotope compositions of methane (δ^13^C_VPDB_ values) during hydrogenotrophic methanogenesis by incubating enriched sediments with carbonate minerals; Figure S10: Time dependence of the concentration of extracted DNA and the concentration of total organic carbon during the incubation of enriched sediment with hydrogen and the carbonate minerals and for control experiments; Figure S11: Time dependence of microbial composition richness and evenness in raw sediment (gray star), hydrogen-enriched sediment prior to carbonate mineral addition, and during the incubation of enriched sediment with hydrogen and the carbonate minerals; Figure S12: The relative abundance of all identified microorganisms at the genus level in the initial sediment sample G, hydrogen-enriched sediment prior to carbonate mineral addition, and after 20-day incubation of enriched sediment with hydrogen and the carbonate minerals or sodium bicarbonate; Figure S13: Time-dependence of the relative abundance of microorganisms in the domain of Archaea at a genus level in raw sediment, hydrogen-enriched sediment prior to carbonate mineral addition, and during the incubation of enriched sediment with hydrogen and the carbonate minerals; Figure S14: Time-dependence of the relative abundance of microorganisms in the domain of Bacteria at a genus level in raw sediment, hydrogen-enriched sediment prior to carbonate mineral addition, and during the incubation of enriched sediment with hydrogen and the carbonate minerals; Figure S15: The relative abundance of *Methanobacterium* at a genus level relative to all identified microbial community members and relative to members in the domain of Archaea and the relative abundance of *Acetobacterium* at a genus level relative to all identified microbial community members and relative to members in the domain of Bacteria; Figure S16: Similarity and dissimilarity of samples based on qualitative phylogenetic beta diversity and quantitative beta diversity measures weighted by taxon abundance; Figure S17: Biogeochemical modeling results of dynamics of the biomass growth of acetogen, methanogen, and sulfate reduction bacteria (SRB) and the thermodynamic potential factor (TPF) of acetogenesis, methanogenesis, and sulfate reduction for hydrogenotrophy with calcite (CaCO_3_) and that for rhodochrosite (MnCO_3_); Figure S18: SEM images showing biofilm development on calcite surfaces over 105 days; Figure S19: Images of biofilm on calcite surfaces by fluorescent imaging of F420 autofluorescence; Figure S20: SEM image of acetogen-calcite association from *Acetobacterium wieringae* pure culture incubation with calcite; Figure S21: Optical photothermal infrared (O-PTIR) spectroscopy results of biofilm on calcite surfaces (PDF)
