# Supplementary material for: Methanogenesis and Acetogenesis in Hydrogenotrophy with Carbonate Minerals: Dependence on Mineral Surface Area, Biofilm Growth, and Microbial Community
Source: Environ Sci Technol. 2025 Aug 12;59(33):17485–95. doi: 10.1021/acs.est.4c14291 (PMC12392440; doi:10.1021/acs.est.4c14291)
Supplement: Supplementary file 1 [file es4c14291_si_001.pdf]

# Supporting Information

# Methanogenesis and Acetogenesis in Hydrogenotrophy with Carbonate Minerals: Dependence on Mineral Surface Area, Biofilm Growth, and Microbial Community

49 Pages, 5 Texts, 9 Tables, 21 Figures

*Yarong Qi[1,2], Sharon Borglin[2], Langlang Li[2,3], Wenming Dong[2], Markus Bill[2], Zhao Hao[2], Céline Pallud[1], Benjamin Gilbert[2,3,*]*

1. Department of Environmental Science, Policy, and Management, University of California, Berkeley, CA, 94720, United States
2. Earth and Environmental Sciences Area, Lawrence Berkeley National Laboratory (LBNL), Berkeley, CA, 94720, United States
3. Department of Earth and Planetary Science, University of California, Berkeley, CA, 94720, United States

*Corresponding author:

bgilbert@lbl.gov

**Supporting Information**

**Text S1**. Calculation of Gibbs free energy change of reaction.
**Text S2**. Biogeochemical modeling with the Geochemist's Workbench (GWB).
**Text S3**. Polymerase Chain Reaction (PCR) of 16S rRNA gene.
**Text S4**. Microbial community analysis on QIIME2.
**Text S5**. Code for microbial community analysis on QIIME2.
**Table S1**. Microbial metabolic processes stimulated by hydrogen.
**Table S2**. The solubility of carbonate minerals at 25℃ and the measured surface area.
**Table S3**. Inorganic medium composition.
**Table S4**. Trace element solution composition.
**Table S5**. Gibbs free energy changes of methane and acetate production by hydrogen oxidation coupled to bicarbonate reduction under standard condition and physiological standard condition.
**Table S6**. Gibbs free energy changes of methane production by hydrogen oxidation coupled to the reduction of carbonate mineral under standard condition and physiological standard condition.
**Table S7**. Gibbs free energy changes of acetate production by of hydrogen oxidation coupled to the reduction of carbonate mineral under standard condition and physiological standard condition.
**Table S8.** The rate law parameters for GWB simulation of microbial growth with hydrogen and calcite ($CaCO_3$) or rhodochrosite ($MnCO_3$).
**Table S9.** The rate law parameters for GWB simulation of carbonate mineral dissolution of calcite ($CaCO_3$) or rhodochrosite ($MnCO_3$).
**Figure S1**. Location and photographs of the coastal sediment collection site.
**Figure S2**. Mineral phase abundance in anaerobic sediment.
**Figure S3**. Time dependence of $H_2$, methane and acetate concentrations in control experiments.
**Figure S4**. Time dependence of the generation of gas-phase hydrogen and methane and aqueous acetate during the incubation of enriched sediment with 10% hydrogen and 20g/L carbonate minerals.
**Figure S5**. Time dependence of (**A**) calculated concentrations of bicarbonate ($HCO_3^-$), (**B**) measured concentrations of inorganic carbon (IC), and (**C**) measured pH during the incubation of enriched sediment with hydrogen and the carbonate minerals.
**Figure S6**. The dynamics of (A) carbonate mineral derived cation in micromoles and (B) cation concentrations in incubations with carbonate minerals.
**Figure S7**. Initial rates and normalized initial rates of hydrogenotrophic (**A**), (**B**), and (**C**) methanogenesis and (**D**), (**E**), and (**F**) acetogenesis correlated to the carbonate mineral solubility (25 ℃, **Table S2**) and the inverse of solubility.
**Figure S8**. The dynamics of free energy changes, $\Delta G_r$, of hydrogenotrophic methanogenesis and acetogenesis calculated based on (**A**) consumption of bicarbonate ions for methane production and (**B**) acetate production (**Table S5**), and (**C**) consumption of carbonate minerals for methane production and (**D**) acetate production (**Tables S6-7)**.
**Figure S9**. Measured time-dependence of carbon isotope compositions of $CH_4$ ($\delta^{13}C_{VPDB}$ values) during hydrogenotrophic methanogenesis by incubating enriched sediments with carbonate minerals.
**Figure S10**. Time dependence of (**A**) the concentration of extracted DNA and (**B**) the concentration of total organic carbon during the incubation of enriched sediment with hydrogen and the carbonate minerals and for control experiments.

**Figure S11**. Time dependence of microbial composition (**A**) richness, and (**B**) evenness in raw sediment (grey star), hydrogen-enriched sediment prior to carbonate mineral addition, and during the incubation of enriched sediment with hydrogen and the carbonate minerals.
**Figure S12**. The relative abundance of all identified microorganisms at the genus level in the initial sediment sample G, hydrogen-enriched sediment prior to carbonate mineral addition, and after 20-day incubation of enriched sediment with hydrogen and the carbonate minerals or sodium bicarbonate.
**Figure S13**. Time-dependence of the relative abundance of microorganisms in the domain of Archaea at a genus level in raw sediment, hydrogen-enriched sediment prior to carbonate mineral addition, and during the incubation of enriched sediment with hydrogen and the carbonate minerals.
**Figure S14**. Time-dependence of the relative abundance of microorganisms in the domain of Bacteria at a genus level in raw sediment, hydrogen-enriched sediment prior to carbonate mineral addition, and during the incubation of enriched sediment with hydrogen and the carbonate minerals.
**Figure S15**. **Top**: The relative abundance of *Methanobacterium* at a genus level (**A**) relative to all identified microbial community members and (**B**) relative to members in the domain of Archaea. **Bottom**: The relative abundance of *Acetobacterium* at a genus level (**C**) relative to all identified microbial community members and (**D**) relative to members in the domain of Bacteria.
**Figure S16**. Similarity and dissimilarity of samples based on (**A**) qualitative phylogenetic beta diversity, and (**B**) quantitative beta diversity measures weighted by taxon abundance.
**Figure S17**. Biogeochemical modeling results of dynamics of (**A**) the biomass growth of acetogen, methanogen, and sulfate reduction bacteria (SRB) and (**B**) the thermodynamic potential factor (TPF) of acetogenesis, methanogenesis, and sulfate reduction for hydrogenotrophy with calcite ($CaCO_3$) and (**C**) and (**D**) that for rhodochrosite ($MnCO_3$).
**Figure S18**. SEM images showing biofilm development on calcite surfaces over 105 days.
**Figure S19**. Images of biofilm on calcite surfaces by fluorescent imaging of $F_{420}$ autofluorescence.
**Figure S20**. SEM image of acetogen-calcite association from *Acetobacterium wieringae* pure culture incubation with calcite.
**Figure S21**. Optical photothermal infrared (O-PTIR) spectroscopy results of biofilm on calcite surfaces.

**Text S1. Calculation of free energy change of reaction with dissolved or mineral carbonate.**

The free energy change $\Delta G_r$ of reaction can be expressed as:

$$\Delta G_r = \Delta G_r^0 + RT\ ln\ Q\ = RT\ ln \frac{Q}{K} < 0 \qquad \textbf{(Equation S1)}$$

where the $\Delta G_r^0$ is the free energy change under standard conditions with pressure of 1 atm, the temperature, T = 298.15K, the aqueous solutions are at 1M concentration and the pH = 0. $K$ is equilibrium constant, $Q$ is the reaction quotient and $R$ is the gas constant (8.314 $J.mol^{-1}.K^{-1}$. To convert $\Delta G_0$ to $\Delta G_0'$ i.e. from standard conditions (pH=0) to biochemical conditions (pH=7) in reactions involving $H^+$ :

$$\Delta G_r^{0\prime} = \Delta G_r^0 + mG'_{f,H^+} \qquad \textbf{(Equation S2)}$$

Where $m$ is the net number of protons in the reaction (negative if more are consumed than produced) and the free energy of formation of a proton at pH 7, $G'_{f,H^+}$ = -39.83 kJ at 25°C.

The results of the calculations are given in the following tables:

**Table S5**. Gibbs free energy changes of hydrogen oxidation coupled to bicarbonate reduction in methanogenesis and acetogenesis under standard conditions.

**Table S6**. Gibbs free energy changes of hydrogenotrophic methanogenesis coupled to carbonate mineral dissolution under standard and physiological conditions.

**Table S7**. Gibbs free energy changes of hydrogenotrophic acetogenesis coupled to carbonate mineral dissolution under standard and physiological conditions.

## Text S2. Biogeochemical modeling with the Geochemist's Workbench.

Biogeochemical modeling was performed using the React module of the Geochemist's Workbench (GWB) using an equilibrium speciation and kinetic rate laws described in the instruction manual.[1] Simulations used the default thermodynamic database, an overview of the modeling approach and inputs are summarized below, and the input files are attached.

### Microbial metabolic modeling

A general metabolic redox reaction is written:

$$\sum_D \upsilon_D D + \sum_{A^+} \upsilon_{A^+} A^+ = \sum_{D^+} \upsilon_{D^+} D^+ + \sum_A \upsilon_A A \quad \textbf{(Equation S3)}$$

where $D$ and $D^+$ are the electron donor reactants and products, respectively, $A^+$ and $A$ are the electron acceptor reactants and products, and $\upsilon$ are the stoichiometric constants. GWB provides a versatile rate law for such metabolic reactions of the form:

$$r_k^{\rightarrow} = n_w k_+ [X] \frac{\prod_j m_j^{P_j}}{\left(K_D \prod_{D^+} m_{D^+}^{P_{D^+}} + \prod_D m_D^{\beta_D}\right)^{P_{KD}} \left(K_A \prod_A m_A^{\beta_A} + \prod_{A^+} m_{A^+}^{P_{A^+}}\right)^{P_{KA}}} \textbf{TPF} \quad \textbf{(Equation S4)}$$

where $r_k^{\rightarrow}$ is the forward reaction rate (moles $s^{-1}$), $n_w$ (kg) is the water mass, [X] (mg $kg^{-1}$) is the biomass concentration and $k_+$ (mol $mg^{-1}$ $s^{-1}$) is the intrinsic reaction rate constant, $m_D$ is the molality of the reduced donor species, *etc*, and TPF is the thermodynamic potential factor, described below.

The GWB rate law expression describes the rate of biomass production as a function of dissolved electron donor and acceptor concentrations. This approach does not distinguish between ATP generation and energy conservation (catabolism) and biosynthesis that utilizes ATP (anabolism), a distinction that has been included in other kinetic modeling approaches.[2] Loss of biomass is described by an exponential decay function ($s^{-1}$).

Following conventional (abiotic) reaction kinetics theory, reaction kinetics of reaction are influenced by reactant concentrations raised to the power of their stoichiometry so that $P_D = \upsilon_D$, *etc*. We further assume that the reactants have no inhibitory effects on the reaction rate and hence $P_{D^+} = P_A = 0$. Because there is no information about higher-order controls on the kinetics we also set $P_{KA} = P_{KA} = 1$.

Thus, for methanogenesis, the metabolic reaction and corresponding kinetic rate law are:

$$H_{2(aq)} + 0.25\ HCO_3^- + 0.25\ H^+ \rightarrow 0.75\ H_2O + 0.25\ CH_{4(aq)} \quad \textbf{(Reaction S1)}$$

$$r_{\vec{k}} = n_w k_+ [X] \frac{m_{H_{2(aq)}} m_{HCO_3^-}^{1/4} m_{H^+}^{1/4}}{\left(K_D + m_{H_{2(aq)}}\right)\left(K_A + m_{HCO_3^-}^{1/4} m_{H^+}^{1/4}\right)} \mathbf{TPF} \qquad \textbf{(Equation S5)}$$

In analogy to enzymatic reactions, the parameters $K_D$ and $K_A$ are "half-saturation constants". Like enzymes, microbes cannot drive metabolic reactions at arbitrarily fast rates even at high reactant concentration, inclusion of half-saturation constants leads to more realistic predictions.

The thermodynamic potential factor (TPF) describes how the reaction rate may be affected by the available free energy from the reaction, $\Delta G_r$, based on departure from equilibrium that is calculated by reactant and product activities. The available free energy is determined from the reaction quotient $Q = \prod_i a^{\nu_i}$ with an adapted form of $\Delta G_r$ used here for the simulation.

Microorganisms use free energy from metabolic processes to generate ATP, which has a free energy of formation $\Delta G_{ATP}$ = 45 kJ mol$^{-1}$, by proton pumping to create a chemiosmotic gradient harnessed by ATPases or by substrate-level phosphorylation. Following Jin and Bethke[3], each metabolic pathway is characterized by $n_{ATP}$, the number of ATP molecules that are generated by a full metabolic reaction, and $\chi$, the number of times that a rate-limiting intermediate step, often interpreted to be proton translocation, occurs. The GWB expression for the TPF is then:

$$\mathbf{TPF} = \left[1 - \left(\frac{Q}{K}\right)^{\frac{1}{\chi}} \exp\left(\frac{-n_{ATP}\Delta G_{ATP}}{\chi R T_K}\right)\right]^{\Omega} \qquad \textbf{(Equation S6)}$$

where in the GWB software, $\chi = \frac{1}{\omega}$, where $\omega$ is called 'order 1' and $\Omega = 1$ is called 'order 2'. The starting metabolic parameters for sulfur reduction and methanogenesis were taken from Jin et al.[4], in a study of ethanol metabolism that included hydrogenotrophic sulfate reduction and methanogenesis. Despite a review of the literature, we were not able to identify most probably metabolic parameters for acetogenesis, as physiological behavior various considerably.[5] Thus, we used the same parameters as for methanogenesis.

**Mineral Dissolution**

Reactions in calcite dissolution and carbonate association:

$CaCO_{3(s)} \rightarrow Ca^{2+} + CO_3^{2-}$ **(Reaction S2)**

$CO_3^{2-} + H_2O \rightarrow OH^- + HCO_3^-$ **(Reaction S3)**

$HCO_3^- + H_2O \rightarrow OH^- + H_2CO_{3(aq)}$ (**Reaction S4**)

Carbonate mineral dissolution was simulated using:

$$r_{\vec{k}} = A_S k_+ \left(1 - \frac{Q}{K}\right) \quad \textbf{(Equation S7)}$$

where $r_{\vec{k}}$ is the reaction rate (mol $s^{-1}$), $A_S$ is surface area ($cm^2$), and $k_+$ is rate constant (mol $cm^{-2}$ $s^{-1}$).

**Methane gas exchange**

The rate of methane exsolution at the gas/fluid interface (mol $s^{-1}$) was calculated using:

$$r_{\vec{k}} = nA_S k_+ \left(f_{gas} - f_{fluid}\right) \quad \textbf{(Equation S8)}$$

where n = 3 g is the mass of the fluid, $A_S$ = 0.03 $cm^2$ $kg^{-1}$ is surface area per mass of fluid, $f$ is gas fugacity, and $k_+$ is rate constant. $f_{gas}$ was fixed at the value for a reservoir in equilibrium with fluid with dissolved methane at $10^{-5}$ mol $kg^{-1}$ and that was set equal to 1 mol $cm^{-2}$ $s^{-1}$ ensuring rapid transfer. The rate of methane production was determined from the rate of methane efflux. The default GWB code considers an infinite gas reservoir and not a fixed volume of gas, and hence the gas phase methane fugacity relevant to a constant headspace vial is not dynamically updated during the simulation. Additional scripting would be required to achieve this (see discussion on GWB Forum). Consequently, potential thermodynamic constraints on methanogenesis as methane is generated are not accurately calculated. However, methanogenesis remained far from equilibrium during the incubations.

## GWB input file for hydrogenotrophy with calcite

```
# React script, saved by BGilbert

data = thermo.tdat verify
conductivity = conductivity-USGS.dat

suppress  (BaO)2^(SiO2)3(c) (BaO)2^SiO2(c) (UO2)3(PO4)2(c) (VO)3(PO4)2(c)
suppress  Acanthite Ag3PO4(c) Akermanite Al(AsO4)(c)
suppress  Al2(SO4)3 Al2(SO4)3^6H2O Alabandite Albite
suppress  "Albite high" "Albite low" Alunite Amesite-14A
suppress  Analc-dehydr Analcime Andalusite Andradite
suppress  Anglesite Anhydrite Annite Anorthite
suppress  Anthophyllite Antigorite Arcanite Arsenopyrite
suppress  BaHPO4(c) BaO^(SiO2)2(c) BaO^SiO2(c) BaS(c)
suppress  BaSiF6(c) Barite Bassanite Bassetite
suppress  Beidellit-Ca Beidellit-H Beidellit-K Beidellit-Mg
suppress  Beidellit-Na Berlinite Bieberite Bloedite
suppress  Boehmite Boltwood-Na Boltwoodite Bornite
suppress  Brezinaite Burkeite "Ca-Al Pyroxene" Ca2Si3O8^5/2H2O
suppress  Ca2SiO4(gamma) Ca2SiO4^7/6H2O Ca3Si2O7^3H2O Ca3SiO5
suppress  Ca4Si3O10^3/2H2O Ca5Si6O17^11/2H2O Ca5Si6O17^21/2H2O Ca5Si6O17^3H2O
suppress  Ca6Si6O18^H2O CaHPO4^2H2O CaSO4^1/2H2O(beta) CaSi2O5^2H2O
suppress  Cattierite Celestite Chalcedony Chalcocite
suppress  Chalcopyrite Chamosite-7A Chloropyromorphite Chrysotile
suppress  Cinnabar Clinochl-14A Clinochl-7A Clinoptil-Ca
suppress  Clinoptil-K Clinoptil-Mg Clinoptil-Na Clinozoisite
suppress  Co(FeO2)2 Co2SiO4 Co3(PO4)2 CoHPO4
suppress  CoS CoSO4(s) CoSO4^3Co(OH)2 CoSO4^6H2O
suppress  CoSO4^H2O Coffinite Cordier^anhy Cordier^hydr
suppress  Corundum Covellite CrS Cristobalite
suppress  Cronstedt-7A Cu3(PO4)2(c) Cu3(PO4)2^3H2O CuFeO2(c)
suppress  Daphnite-14A Daphnite-7A Dawsonite Diaspore
suppress  Diopside Enstatite Epidote Epidote-ord
suppress  Epsomite Eu2(SO4)3^8H2O EuS EuSO4
suppress  Eucryptite Fayalite Fe(OH)2(ppd) Fe(OH)3(ppd)
suppress  Fe2(SO4)3(c) FeCr2O4 FeF2(c) FeF3(c)
suppress  FeO(c) FeSO4(c) FeSe FeSe2
suppress  FeV2O4(c) Ferrite-2-Ca Ferrite-Ca Ferrite-Cu
suppress  Ferrite-Mg Ferrite-Zn Ferrosilite Fluorapatite
suppress  Forsterite Galena Gehlenite Gibbsite
suppress  Goethite Greenalite Grossular Gypsum
suppress  Haiweeite Hedenbergite Hematite Hercynite
suppress  Heulandite Hexahydrite Hinsdalite Hydroxyapatite
suppress  Hydroxypyromorphite Illite Jadeite Jarosite-K
suppress  Jarosite-Na Kainite Kalsilite Kasolite
suppress  Kieserite Kyanite Larnite Laumontite
suppress  Lawrencite Lawsonite Leonhardtite Linnaeite
suppress  MHSH(Mg1.5) Magnetite Margarite "Maximum Microcline"
suppress  Melanterite Mercallite Merwinite Metacinnabar
suppress  MgSO4(c) Minnesotaite Mirabilite Misenite
```

```
suppress  Mn3(PO4)2(c) MnHPO4(c) MnSO4(c) Molysite
suppress  Monticellite Mordenite-K Mordenite-Na Muscovite
suppress  Na2Si2O5 Na2SiO3 Na3H(SO4)2 Na4SiO4
suppress  Na6Si2O7 NaFeO2(c) Nepheline Ni2P2O7
suppress  Ni2SiO4 Ni3(PO4)2 Ni3S2 Ni3S4
suppress  NiFe2O4 NiS NiS2 NiSO4(s)
suppress  NiSO4^6H2O NiSO4^7H2O Ningyoite Nontronit-Ca
suppress  Nontronit-K Nontronit-Mg Nontronit-Na Np(HPO4)2(s)
suppress  Orpiment Paragonite Pargasite Pb3(PO4)2(c)
suppress  Pb4O(PO4)2(c) PbHPO4(c) Pentahydrite Petalite
suppress  Phengite Phlogopite Plumbogummite Prehnite
suppress  Przhevalskite Pseudowollastonite PuO2HPO4(c) Pyrite
suppress  Pyrophyllite Quartz RaSO4 Rankinite
suppress  Realgar Rhodonite Ripidolit-14A Ripidolit-7A
suppress  RuS2(s) S-- S2-- S3--
suppress  S4-- S5-- S6-- Saleeite
suppress  "Sanidine high" Saponite-Ca Saponite-H Saponite-K
suppress  Saponite-Mg Saponite-Na Scorodite Sepiolite
suppress  Siderite Sillimanite Sklodowskite Smectite-Reykjanes
suppress  Smectite-high-Fe-Mg Smectite-low-Fe-Mg Sn(SO4)2(c) Sn2S3
suppress  Sn3S4 SnS SnSO4 Soddyite
suppress  Sphalerite Spinel Spodumene-a Sr-Autunite
suppress  Sr2SiO4(c) SrHPO4(c) SrS(c) SrSiO3(c)
suppress  Strengite Sulfur-Rhmb Talc Tc2S7(s)
suppress  TcS2(s) TcS3(s) Tephroite Th(SO4)2(c)
suppress  Th2S3 Th7S12 ThS2 Thenardite
suppress  Torbernite Tremolite Tridymite Troilite
suppress  Tsumebite U(HPO4)2^4H2O U(SO4)2(c) UO2HPO4(c)
suppress  UO2SO4(c) UO2SO4^(2.5H2O) UO2SO4^(3.5H2O) UO2SO4^3H2O
suppress  UO2SO4^H2O Uranocircite Uranophane Vivianite
suppress  Wairakite Weeksite Whitlockite Wollastonite
suppress  Wurtzite Wustite Zn3(PO4)2^4H2O Zoisite

time start = 0 day, end = 20 day

temperature = 25 C

decouple CH3COO-
decouple CH4(aq)
decouple H2(aq)
decouple HS-
decouple N2(aq)
decouple NH4+
decouple NO2-

H2O        = .003 free kg

swap H2(g) for H2(aq)

H2(g)      = .1 fugacity
pH         = 7.4
```

```
Cl-         = 20 mmol/kg
balance on Cl-
Na+         = 20 mmol/kg
swap Calcite for Ca++
Calcite     = 20 free g/kg
O2(aq)      = 1e-10 mmol/kg
HCO3-       = 2 mmol/kg
CH4(aq)     = 1e-5 mmol/kg
SiO2(aq)    = 4 umol/kg
Br-         = .01 free mmol/kg
CH3COO-     = 1e-5 mmol/kg
Al+++       = .5 umol/kg
K+          = 25 umol/kg
swap KHPO4- for HPO4--
KHPO4-      = 5.1 mmol/l
NH4+        = 10 mmol/l
HS-         = 1e-5 mmol/l
SO4--       = .15 mmol/l
Fe++        = .1 mmol/l

kinetic Calcite 100 mmol/kg rate_con = 4.2e-12 surface = 500

kinetic microbe-Methanogen rxn = "H2(aq)  + .25 HCO3-  + .25 H+  -> .75 H2O  + .25 CH4(aq)"
biomass = 1 rate_con = 1e-10 KA = 4.7e-6 KD = 5e-6 apower(H2(aq)) = 1 apower(HCO3-) = .25
apower(H+) = .25 apowerD(H2(aq)) = 1 apowerA(HCO3-) = .25 apowerA(H+) = .25 ATP_energy = -45
ATP_number = .25 growth_yield = 1080 decay_con = 1e-7 order1 = 6

kinetic microbe-Acetogen rxn = "H2(aq)  + .5 HCO3-  + .25 H+  -> H2O  + .25 CH3COO-" biomass = 1
rate_con = 1.5e-10 KA = 4.7e-6 KD = 5e-6 apower(H2(aq)) = 1 apower(HCO3-) = .5 apower(H+) = .25
apowerD(H2(aq)) = 1 apowerA(HCO3-) = .5 apowerA(H+) = .25 ATP_energy = -45 ATP_number = .25
growth_yield = 1000 decay_con = 1e-7 order1 = 6

kinetic microbe-SRB rxn = "H2(aq)  + .25 H+  + .25 SO4--  -> .25 HS-  + H2O" biomass = 1 rate_con =
1e-9 KA = 1.1e-6 KD = 3.9e-5 apower(H2(aq)) = 1 apower(SO4--) = .25 apower(H+) = .25
apowerD(H2(aq)) = 1 apowerA(SO4--) = .25 apowerA(H+) = .25 ATP_energy = -45 ATP_number = .71
growth_yield = 2190 decay_con = 1e-7 order1 = .667

fix fugacity of H2(g)

kinetic CH4(g) rate_con = 1 surface = .03
```

**GWB input file for hydrogenotrophy with rhodochrosite**

```
# React script, saved by BGilbert

data = thermo.tdat verify
conductivity = conductivity-USGS.dat

suppress  (BaO)2^(SiO2)3(c) (BaO)2^SiO2(c) (UO2)3(PO4)2(c) (VO)3(PO4)2(c)
suppress  Acanthite Ag3PO4(c) Akermanite Al(AsO4)(c)
suppress  Al2(SO4)3 Al2(SO4)3^6H2O Alabandite Albite
suppress  "Albite high" "Albite low" Alunite Amesite-14A
suppress  Analc-dehydr Analcime Andalusite Andradite
suppress  Anglesite Anhydrite Annite Anorthite
suppress  Anthophyllite Antigorite Arcanite Arsenopyrite
suppress  BaHPO4(c) BaO^(SiO2)2(c) BaO^SiO2(c) BaS(c)
suppress  BaSiF6(c) Barite Bassanite Bassetite
suppress  Beidellit-Ca Beidellit-H Beidellit-K Beidellit-Mg
suppress  Beidellit-Na Berlinite Bieberite Bloedite
suppress  Boehmite Boltwood-Na Boltwoodite Bornite
suppress  Brezinaite Burkeite "Ca-Al Pyroxene" Ca2Si3O8^5/2H2O
suppress  Ca2SiO4(gamma) Ca2SiO4^7/6H2O Ca3Si2O7^3H2O Ca3SiO5
suppress  Ca4Si3O10^3/2H2O Ca5Si6O17^11/2H2O Ca5Si6O17^21/2H2O Ca5Si6O17^3H2O
suppress  Ca6Si6O18^H2O CaHPO4^2H2O CaSO4^1/2H2O(beta) CaSi2O5^2H2O
suppress  Cattierite Celestite Chalcedony Chalcocite
suppress  Chalcopyrite Chamosite-7A Chloropyromorphite Chrysotile
suppress  Cinnabar Clinochl-14A Clinochl-7A Clinoptil-Ca
suppress  Clinoptil-K Clinoptil-Mg Clinoptil-Na Clinozoisite
suppress  Co(FeO2)2 Co2SiO4 Co3(PO4)2 CoHPO4
suppress  CoS CoSO4(s) CoSO4^3Co(OH)2 CoSO4^6H2O
suppress  CoSO4^H2O Coffinite Cordier^anhy Cordier^hydr
suppress  Corundum Covellite CrS Cristobalite
suppress  Cronstedt-7A Cu3(PO4)2(c) Cu3(PO4)2^3H2O CuFeO2(c)
suppress  Daphnite-14A Daphnite-7A Dawsonite Diaspore
suppress  Diopside Enstatite Epidote Epidote-ord
suppress  Epsomite Eu2(SO4)3^8H2O EuS EuSO4
suppress  Eucryptite Fayalite Fe(OH)2(ppd) Fe(OH)3(ppd)
suppress  Fe2(SO4)3(c) FeCr2O4 FeF2(c) FeF3(c)
suppress  FeO(c) FeSO4(c) FeSe FeSe2
suppress  FeV2O4(c) Ferrite-2-Ca Ferrite-Ca Ferrite-Cu
suppress  Ferrite-Mg Ferrite-Zn Ferrosilite Fluorapatite
suppress  Forsterite Galena Gehlenite Gibbsite
suppress  Goethite Greenalite Grossular Gypsum
suppress  Haiweeite Hedenbergite Hematite Hercynite
suppress  Heulandite Hexahydrite Hinsdalite Hydroxyapatite
suppress  Hydroxypyromorphite Illite Jadeite Jarosite-K
suppress  Jarosite-Na Kainite Kalsilite Kasolite
suppress  Kieserite Kyanite Larnite Laumontite
suppress  Lawrencite Lawsonite Leonhardtite Linnaeite
suppress  MHSH(Mg1.5) Magnetite Margarite "Maximum Microcline"
suppress  Melanterite Mercallite Merwinite Metacinnabar
```

```
suppress  MgSO4(c) Minnesotaite Mirabilite Misenite
suppress  Mn3(PO4)2(c) MnHPO4(c) MnSO4(c) Molysite
suppress  Monticellite Mordenite-K Mordenite-Na Muscovite
suppress  Na2Si2O5 Na2SiO3 Na3H(SO4)2 Na4SiO4
suppress  Na6Si2O7 NaFeO2(c) Nepheline Ni2P2O7
suppress  Ni2SiO4 Ni3(PO4)2 Ni3S2 Ni3S4
suppress  NiFe2O4 NiS NiS2 NiSO4(s)
suppress  NiSO4^6H2O NiSO4^7H2O Ningyoite Nontronit-Ca
suppress  Nontronit-K Nontronit-Mg Nontronit-Na Np(HPO4)2(s)
suppress  Orpiment Paragonite Pargasite Pb3(PO4)2(c)
suppress  Pb4O(PO4)2(c) PbHPO4(c) Pentahydrite Petalite
suppress  Phengite Phlogopite Plumbogummite Prehnite
suppress  Przhevalskite Pseudowollastonite PuO2HPO4(c) Pyrite
suppress  Pyrophyllite Quartz RaSO4 Rankinite
suppress  Realgar Rhodonite Ripidolit-14A Ripidolit-7A
suppress  RuS2(s) S-- S2-- S3--
suppress  S4-- S5-- S6-- Saleeite
suppress  "Sanidine high" Saponite-Ca Saponite-H Saponite-K
suppress  Saponite-Mg Saponite-Na Scorodite Sepiolite
suppress  Siderite Sillimanite Sklodowskite Smectite-Reykjanes
suppress  Smectite-high-Fe-Mg Smectite-low-Fe-Mg Sn(SO4)2(c) Sn2S3
suppress  Sn3S4 SnS SnSO4 Soddyite
suppress  Sphalerite Spinel Spodumene-a Sr-Autunite
suppress  Sr2SiO4(c) SrHPO4(c) SrS(c) SrSiO3(c)
suppress  Strengite Sulfur-Rhmb Talc Tc2S7(s)
suppress  TcS2(s) TcS3(s) Tephroite Th(SO4)2(c)
suppress  Th2S3 Th7S12 ThS2 Thenardite
suppress  Torbernite Tremolite Tridymite Troilite
suppress  Tsumebite U(HPO4)2^4H2O U(SO4)2(c) UO2HPO4(c)
suppress  UO2SO4(c) UO2SO4^(2.5H2O) UO2SO4^(3.5H2O) UO2SO4^3H2O
suppress  UO2SO4^H2O Uranocircite Uranophane Vivianite
suppress  Wairakite Weeksite Whitlockite Wollastonite
suppress  Wurtzite Wustite Zn3(PO4)2^4H2O Zoisite

time start = 0 day, end = 25 day

temperature = 25 C

decouple CH3COO-
decouple CH4(aq)
decouple H2(aq)
decouple HS-
decouple N2(aq)
decouple NH4+
decouple NO2-

H2O         = .003 free kg

swap H2(g) for H2(aq)

H2(g)       = .1 fugacity
```

```
pH          = 6.9
Cl-         = 20 mmol/kg
balance on Cl-
Na+         = 20 mmol/kg
swap Calcite for Ca++
Calcite     = 20 free mg
swap Rhodochrosite for Mn++
Rhodochrosite = 20 free g/kg
O2(aq)      = 1e-10 mmol/kg
HCO3-       = 2 mmol/kg
CH4(aq)     = 1e-5 mmol/kg
SiO2(aq)    = 4 umol/kg
Br-         = .01 free mmol/kg
CH3COO-     = 1e-5 mmol/kg
Al+++       = .5 umol/kg
K+          = 25 umol/kg
swap KHPO4- for HPO4--
KHPO4-      = 5.1 mmol/l
NH4+        = 10 mmol/l
HS-         = 1e-5 mmol/l
SO4--       = .15 mmol/l
Fe++        = .1 mmol/l

kinetic Rhodochrosite 100 mmol/kg rate_con = 4.2e-12 surface = 5000

kinetic microbe-Methanogen rxn = "H2(aq)  + .25 HCO3-  + .25 H+  -> .75 H2O  + .25 CH4(aq)"
biomass = 1 rate_con = 1.76e-10 KA = 4.7e-6 KD = 5e-6 apower(H2(aq)) = 1 apower(HCO3-) = .25
apower(H+) = .25 apowerD(H2(aq)) = 1 apowerA(HCO3-) = .25 apowerA(H+) = .25 ATP_energy = -45
ATP_number = .25 growth_yield = 1080 decay_con = 1e-7 order1 = 6

kinetic microbe-Acetogen rxn = "H2(aq)  + .5 HCO3-  + .25 H+  -> H2O  + .25 CH3COO-" biomass = 1
rate_con = 1.2e-9 KA = 4.7e-6 KD = 5e-6 apower(H2(aq)) = 1 apower(HCO3-) = .5 apower(H+) = .25
apowerD(H2(aq)) = 1 apowerA(HCO3-) = .5 apowerA(H+) = .25 ATP_energy = -45 ATP_number = .2
growth_yield = 1000 decay_con = 1e-7 order1 = 6

kinetic microbe-SRB rxn = "H2(aq)  + .25 H+  + .25 SO4--  -> .25 HS-  + H2O" biomass = 1 rate_con =
1e-9 KA = 1.1e-6 KD = 3.9e-5 apower(H2(aq)) = 1 apower(SO4--) = .25 apower(H+) = .25
apowerD(H2(aq)) = 1 apowerA(SO4--) = .25 apowerA(H+) = .25 ATP_energy = -45 ATP_number = .71
growth_yield = 2190 decay_con = 1e-7 order1 = .667

fix fugacity of H2(g)

kinetic CH4(g) rate_con = 1 surface = .03
```

**Text S3. Polymerase Chain Reaction (PCR) of 16S rRNA gene.**

The V4–V5 region of 16S rRNA gene of archaeal and bacterial genomic DNA (gDNA) was amplified with a primer pair 340F/806R (5′-CCCTAYGGGGYGCASCAG-3′ and 5′-GGACTACHVGGGTWTCTAAT-3′) [6, 7] with barcoded 806R according to a standard 16S PCR protocol. Briefly, 25μL solution mixture with 5ng gDNA *per* reaction and 200nmol/L primer was applied for PCR in a T100™ Thermal Cycler with a temperature profile of 95°C for 3 min as the first step, followed by a step 2 with 30 cycles of 95°C for 45s, 50°C for 60s, and 72°C for 90s, and a final step of elongation at 72°C for 10 min before an infinite storage step at 4°C. PCR products were purified and diluted to a concentration of 5ng/μL. The purified products with 10ng of DNA of each sample were combined in a 1.5 mL Eppendorf tube to get a tube of pooled DNA sample (8nM, or 2-5 ng/μL) for amplicon sequencing.

**Text S4. Microbial community analysis on QIIME2.**

The community composition and diversity were analyzed on QIIME2 platform. The paired-end data from the sequencing facility comes demultiplexed and barcodes removed. The adapter-trimmed reads were processed using QIIME2 (version 2023.9.1).[8] Briefly, the reads data quality was assessed with FastQC (version 0.12.1)[9] and summarized with MultiQC (version 1.14)[10] before the sequences were denoised and truncated to 200 bases for both the forward and reverse reads using the QIIME2 plugin DADA2 (version q2-dada2 v2021.2.0).[11] The resultant sequences were assigned with taxonomy using the plugin feature-classifier with a pre-fitted or trained sklearn-based taxonomy classifier.[12] Optionally, this classifier can be trained against the 16S rRNA gene reference database SILVA (release 138, 27.08.2020). The alpha diversity indicating the richness and abundance across samples, commonly referred to as evenness (e.g. Shannon), was analyzed with the diversity plugin. Relative abundance of taxa in samples and the principal component analysis (PCoA) was analyzed based on the counts of clean sequence reads according to QIIME2 tutorial.

**Text S5. Code for microbial community analysis on QIIME2.**

```
# Import data
qiime tools import \
    --type 'SampleData[PairedEndSequencesWithQuality]' \
    --input-path /Users/Directory/raw_data \
    --input-format CasavaOneEightSingleLanePerSampleDirFmt \
    --output-path /Users/Directory/demux_paired_end.qza
# Summarize data and visualization
qiime demux summarize \
   --i-data demux_paired_end.qza \
   --o-visualization demux_paired_end.qzv
# Trimming and Denoising
qiime dada2 denoise-paired \
   --i-demultiplexed-seqs demux_paired_end.qza \
   --p-trunc-len-f 200 \
   --p-trunc-len-r 200 \
   --o-representative-sequences rep-seqs.qza \
   --o-table rep-seqs-table.qza \
   --o-denoising-stats rep-seqs-denoising-stats.qza\
# To visualize the representative sequences:
qiime feature-table tabulate-seqs \
   --i-data rep-seqs.qza \
   --o-visualization rep-seqs.qzv
# Summarize your filtered/denoised data
qiime feature-table summarize \
  --i-table rep-seqs-table.qza \
  --m-sample-metadata-file metadata.txt \
  --o-visualization rep-seqs-table.qzv \
# Train classifier (Optional)
# Extract reference sequences
qiime feature-classifier extract-reads \
   --i-sequences silva-138-99-nb-classifier.qza \
   --p-f-primer CCCTAYGGGGYGCASCAG \
   --p-r-primer GGACTACHVGGGTWTCTAAT \
   --p-max-length 505 \
   --p-min-length 250 \
   --o-reads ref-seqs.qza
 # Trained classifier
qiime feature-classifier fit-classifier-naive-bayes \
   --i-reference-reads ref-seqs.qza \
   --i-reference-taxonomy silva-138-99-tax.qza \
   --o-classifier classifier_naive_bayes.qza
# Classify reads
qiime feature-classifier classify-sklearn \
   --i-classifier classifier_naive_bayes.qza \
   --i-reads rep-seqs.qza \
   --o-classification taxonomy_trained.qza
qiime metadata tabulate \
   --m-input-file taxonomy_trained.qza \
   --o-visualization taxonomy_trained.qzv
```

qiime taxa barplot --i-table rep-seqs-table.qza --i-taxonomy taxonomy_trained.qza --m-metadata-file metadata.txt --o-visualization taxa-bar-plots-trained.qzv

**#Diversity analysis**

```
qiime diversity alpha-rarefaction \
  --i-table rep-seqs-table.qza \
  --i-phylogeny rooted-tree.qza \
  --p-max-depth 862441 \
  --m-metadata-file metadata.txt \
  --o-visualization alpha-rarefaction.qzv
# calculate and explore diversity metrics
qiime diversity core-metrics-phylogenetic \
  --i-phylogeny rooted-tree.qza \
  --i-table rep-seqs-table.qza \
  --p-sampling-depth 4000 \
  --m-metadata-file metadata.txt \
  --output-dir core-metrics-results
```

**# alpha diversity**

```
qiime diversity alpha-group-significance \
  --i-alpha-diversity core-metrics-results/faith_pd_vector.qza \
  --m-metadata-file metadata.txt \
  --o-visualization core-metrics-results/faith-pd-group-significance.qzv
qiime diversity alpha-group-significance \
  --i-alpha-diversity core-metrics-results/evenness_vector.qza \
  --m-metadata-file metadata.txt \
  --o-visualization core-metrics-results/evenness-group-significance.qzv
qiime diversity alpha-group-significance \
  --i-alpha-diversity core-metrics-results/shannon_vector.qza \
  --m-metadata-file metadata.txt \
  --o-visualization core-metrics-results/shannon_group-significance.qzv
```

**# PCoA**

```
qiime metadata tabulate \
    --m-input-file unweighted_unifrac_pcoa_results.qza \
    --o-visualization unweighted_unifrac_pcoa_results.qza.qzv
qiime metadata tabulate \
    --m-input-file bray_curtis_pcoa_results.qza \
    --o-visualization bray_curtis_pcoa_results.qza.qzv
qiime metadata tabulate \
    --m-input-file weighted_unifrac_pcoa_results.qza \
    --o-visualization weighted_unifrac_pcoa_results.qza.qzv
```

**Table S1.** Microbial metabolic processes stimulated by hydrogen.

| Process | Reaction | $\Delta G_r^0$(kJ/mol-$H_2$) |
|---|---|---|
| *Inorganics reduction* | | |
| Aerobic respiration | $2H_2 + O_2 \leftrightarrow 2H_2O$ | -237.3[13], -237.2[14], -237[15] |
| Denitrification | $5H_2 + 2NO_3^- + 2H^+ \leftrightarrow N_2 + 6H_2O$ | -240.1[14], -224[13, 15], -172.9[16] |
| Nitrate reduction | $H_2 + NO_3^- \leftrightarrow NO_2^- + H_2O$ | -163.2[13] |
| Ammonification | $4H_2 + NO_3^- + 2H^+ \leftrightarrow NH_4^+ + 3H_2O$ | -150[15], -149.9[17] |
| Nitrite reduction | $3H_2 + 2NO_2^- + 2H^+ \leftrightarrow N_2 + 4H_2O$ | -265[13] |
| Nitrous oxide reduction | $H_2 + N_2O \leftrightarrow N_2 + H_2O$ | -341.4[13] |
| Nitrogen fixation | $3H_2 + N_2 + 2H^+ \leftrightarrow 2NH_4^+$ | -26.7[13] |
| Iron (III) reduction | $H_2 + 2FeOOH + 4H^+ \leftrightarrow 2Fe^{2+} + 4H_2O$ | -182.5[14] |
| Manganese (IV) reduction | $H_2 + MnO_2 \leftrightarrow Mn(OH)_2$ | -163[15] |
| Arsenate reduction | $H_2 + HA_sO_4^{2-} + 2H^+ \leftrightarrow H_3A_sO_3 + H_2O$ | -162.4[14] |
| Chromate reduction | $3H_2 + 2CrO_4^{2-} + 10H^+ \leftrightarrow 2Cr^{3+} + 8H_2O$ | -90.04[16] |
| Sulfate reduction | $4H_2 + SO_4^{2-} + H^+ \leftrightarrow HS^- + 4H_2O$ | -57[18], -48.0[14]; -38[13] |
| Sulfite reduction | $3H_2 + SO_3^{2-} + H^+ \leftrightarrow HS^- + 3H_2O$ | -57.2[13] |
| Thiosulfate reduction | $4H_2 + S_2O_3^{2-} \leftrightarrow 2HS^- + 3H_2O$ | -43.5[15] |
| Sulfur reduction | $H_2 + S \leftrightarrow HS^- + H^+$ | -27.9[13, 15], -27.8[17] |
| Methanogenesis | $4H_2 + HCO_3^- + H^+ \leftrightarrow CH_4 + 3H_2O$ | -57.3[19], -47.3 (this study), -43.9[14], -34[15], -33.9[13] |
| Acetogenesis | $4H_2 + 2HCO_3^- + H^+ \leftrightarrow CH_3COO^- + 4H_2O$ | -43.6 (this study), -36.1[14], 26.15[13], -26.1[17] |
| Methanol formation | $3H_2 + HCO_3^- + H^+ \leftrightarrow CH_3OH + 2H_2O$ | -7.67[13] |
| Formate formation | $H_2 + HCO_3^- \leftrightarrow HCOO^- + H_2O$ | -1.3[13] |
| *Organics reduction* | | |
| Formate reduction to methane | $3H_2 + HCOO^- + H^+ \leftrightarrow CH_4 + 2H_2O$ | -44.8[13] |
| Formate reduction to methanol | $2H_2 + HCOO^- + H^+ \leftrightarrow CH_3OH + H_2O$ | -10.9[13] |
| Methanol reduction to methane | $H_2 + CH_3OH \leftrightarrow CH_4 + H_2O$ | -112.5[13] |

**Table S2**. The solubility of carbonate minerals at 25℃ and the measured surface area.

| Name | Formula | $K_{sp}$ | Solubility (μmol/L) | Measured surface area ($m^2/g$) |
|---|---|---|---|---|
| Calcium carbonate (calcite) | $CaCO_3$ | $3.36\times10^{-9}$ | 58.0 | 0.52 ± 0.11 |
| Barium carbonate | $BaCO_3$ | $2.58\times10^{-9}$ | 50.8 | 0.52 ± 0.14 |
| Strontium carbonate | $SrCO_3$ | $5.60\times10^{-10}$ | 23.7 | 4.88 ± 0.16 |
| Manganese(II) carbonate | $MnCO_3$ | $2.24\times10^{-11}$ | 4.7 | 9.78 ± 0.41 |

**Table S3**. Inorganic medium composition.[20]

| Reagent | Chemical Formula | Concentration (g/L) |
|---|---|---|
| Potassium dihydrogen phosphate | $KH_2PO_4$ | 0.27 |
| Disodium hydrogen phosphate dodecahydrate | $Na_2HPO_4 \cdot 12H_2O$ | 1.12 |
| Ammonium chloride | $NH_4Cl$ | 0.53 |
| Calcium chloride dihydrate | $CaCl_2 \cdot 2H_2O$ | 0.075 |
| Magnesium chloride hexahydrate | $MgCl_2 \cdot 6H_2O$ | 0.10 |
| Iron(II) chloride tetrahydrate | $FeCl_2 \cdot 4H_2O$ | 0.02 = 100 μM |
| Manganese chloride tetrahydrate | $MnCl_2 \cdot 4H_2O$ | 0.05 = 250 μM |
| Sodium sulfide nonahydrate | $Na_2S \cdot 9H_2O$ | 0.1 |
| Stock solution of trace elements (**Table S4**) | | 10 ml |

**Table S4**. Trace element solution composition.[21]

| Reagent | Chemical Formula | Concentration (g/L) |
|---|---|---|
| Manganese chloride tetrahydrate | $MnCl_2·4H_2O$ | 0.05 |
| Boric acid | $H_3BO_3$ | 0.005 |
| Zinc chloride | $ZnCl_2$ | 0.005 |
| Copper chloride | $CuCl_2$ | 0.003 |
| Disodium molybdate dihydrate | $Na_2MoO_4·2H_2O$ | 0.001 |
| Cobalt chloride hexahydrate | $CoCl_2·6H_2O$ | 0.1 |
| Nickel chloride hexahydrate | $NiCl_2·6H_2O$ | 0.01 |
| Disodium selenite | $Na_2SeO_3$ | 0.005 |
| Disodium tungstate | $Na_2WO_4·2H_2O$ | 0.002 |

**Table S5**. Gibbs free energy changes of methane and acetate production by hydrogen oxidation coupled to bicarbonate reduction under standard condition (1M, 25℃, 1 atm, pH=0) and physiological standard condition (1M, 25℃, 1 atm, pH=7).

| **Reaction** | $\Delta G_r^0$**(kJ/mol-H$_2$)** | $\Delta G_r^{0\prime}$**(kJ/mol-H$_2$)** |
|---|---|---|
| $4H_2 + HCO_{3\,(aq)}^- + H^+ \leftrightarrow CH_4 + 3H_2O$ | -57.3 | -47.3 |
| $4H_2 + 2HCO_{3\,(aq)}^- + H^+ \leftrightarrow CH3COO_{(aq)}^- + 4H_2O$ | -53.6 | -43.6 |

**Table S6**. Gibbs free energy changes of methane production by hydrogen oxidation coupled to the reduction of carbonate mineral controlled electron acceptor under standard condition (1M, 25℃, 1 atm, pH=0) and physiological standard condition (1M, 25℃, 1 atm, pH=7).

| **Reaction** | $\Delta G_r^0$**(kJ/mol-H$_2$)** | $\Delta G_r^{0\prime}$**(kJ/mol-H$_2$)** |
|---|---|---|
| $4H_2 + CaCO_{3(s)} + 2H^+ \leftrightarrow Ca^{2+} + CH_4 + 3H_2O$ | -46.7 | -26.8 |
| $4H_2 + BaCO_{3(s)} + 2H^+ \leftrightarrow Ba^{2+} + CH_4 + 3H_2O$ | -46.3 | -26.4 |
| $4H_2 + SrCO_{3(s)} + 2H^+ \leftrightarrow Sr^{2+} + CH_4 + 3H_2O$ | -63.4 | -43.5 |
| $4H_2 + MnCO_{3(s)} + 2H^+ \leftrightarrow Mn^{2+} + CH_4 + 3H_2O$ | -44.6 | -24.6 |

**Table S7**. Gibbs free energy changes of acetate production by of hydrogen oxidation coupled to the reduction of carbonate mineral controlled electron acceptor under standard condition (1M, 25℃, 1 atm, pH=0) and physiological standard condition (1M, 25℃, 1 atm, pH=7).

| **Reaction** | $\Delta G_r^0$**(kJ/mol-H$_2$)** | $\Delta G_r^{0\prime}$**(kJ/mol-H$_2$)** |
|---|---|---|
| $4H_{2(g)} + 2CaCO_{3(s)} + 3H^+_{(aq)} \leftrightarrow 2Ca^{2+}_{(aq)} + CH3COO^-_{(aq)} + 4H_2O_{(l)}$ | -41.9 | -11.9 |
| $4H_{2(g)} + 2BaCO_{3(s)} + 3H^+_{(aq)} \leftrightarrow 2Ba^{2+}_{(aq)} + CH3COO^-_{(aq)} + 4H_2O_{(l)}$ | -41.1 | -11.1 |
| $4H_{2(g)} + 2SrCO_{3(s)} + 3H^+_{(aq)} \leftrightarrow 2Sr^{2+}_{(aq)} + CH3COO^-_{(aq)} + 4H_2O_{(l)}$ | -75.3 | -45.3 |
| $4H_{2(g)} + 2MnCO_{3(s)} + 3H^+_{(aq)} \leftrightarrow 2Mn^{2+}_{(aq)} + CH3COO^-_{(aq)} + 4H_2O_{(l)}$ | -37.6 | -7.7 |

**Table S8.** The rate law parameters for GWB simulation of microbial growth with hydrogen and calcite ($CaCO_3$) or rhodochrosite ($MnCO_3$). Values that were adjusted in this study are shown in **bold**.

| | Kinetic Parameters | | | | | Thermodynamic Parameters | |
|---|---|---|---|---|---|---|---|
| | $k_+$ mol mg$^{-1}$ s$^{-1}$ | $K_D$ molal | $K_A$ molal | Y g mol$^{-1}$ | D s$^{-1}$ | $n_{ATP}$ | $\chi$ |
| **Sulfate reduction** | | | | | | | |
| $H_{2(aq)} + 0.25\ SO_4^{2-} + 0.25\ H^+ \rightarrow H_2O + 0.25\ HS^-$ | | | | | | | |
| *Jin et al.* | $1 \times 10^{-9}$ | $1.1 \times 10^{-6}$ | $3.9 \times 10^{-6}$ | 2.19 | $1 \times 10^{-7}$ | 0.25 | 1.5 |
| calcite | $\mathbf{1 \times 10^{-9}}$ | $1.1 \times 10^{-6}$ | $3.9 \times 10^{-6}$ | 2.19 | $1 \times 10^{-7}$ | **0.71** | 1.5 |
| rhodochrosite | $\mathbf{1 \times 10^{-9}}$ | $1.1 \times 10^{-6}$ | $3.9 \times 10^{-6}$ | 2.19 | $1 \times 10^{-7}$ | **0.71** | 1.5 |
| **Hydrogenotrophic methanogenesis** | | | | | | | |
| $H_{2(aq)} + 0.25\ HCO_3^- + 0.25\ H^+ \rightarrow 0.75\ H_2O + 0.25\ CH_{4(aq)}$ | | | | | | | |
| *Jin et al.* | $2 \times 10^{-10}$ | $5 \times 10^{-6}$ | $4.7 \times 10^{-6}$ | 1.08 | $1 \times 10^{-7}$ | 0.25 | 6 |
| calcite | $\mathbf{0.9 \times 10^{-10}}$ | $5 \times 10^{-6}$ | $4.7 \times 10^{-6}$ | 1.08 | $1 \times 10^{-7}$ | 0.25 | 6 |
| rhodochrosite | $\mathbf{1.76 \times 10^{-10}}$ | $5 \times 10^{-6}$ | $4.7 \times 10^{-6}$ | 1.08 | $1 \times 10^{-7}$ | 0.25 | 6 |
| **Hydrogenotrophic acetogenesis** | | | | | | | |
| $H_{2(aq)} + 0.5\ HCO_3^- + 0.25\ H^+ \rightarrow H_2O + 0.25\ CH_3CO_2^-$ | | | | | | | |
| calcite | $\mathbf{1.5 \times 10^{-10}}$ | $5 \times 10^{-6}$ | $4.7 \times 10^{-6}$ | 1.08 | $1 \times 10^{-7}$ | 0.25 | 6 |
| rhodochrosite | $\mathbf{12.0 \times 10^{-10}}$ | $5 \times 10^{-6}$ | $4.7 \times 10^{-6}$ | 1.08 | $1 \times 10^{-7}$ | 0.25 | 6 |

**Table S9.** The rate law parameters for GWB simulation of carbonate mineral dissolution of calcite ($CaCO_3$) or rhodochrosite ($MnCO_3$).

| **Mineral** | **Surface Area ($cm^2$ $g^{-1}$)** | **Rate constant (mol $cm^{-2}$ $s^{-1}$)** |
|---|---|---|
| calcite | 500 | $4.2 \times 10^{-12}$ |
| rhodochrosite | 5000 | $4.2 \times 10^{-12}$ |

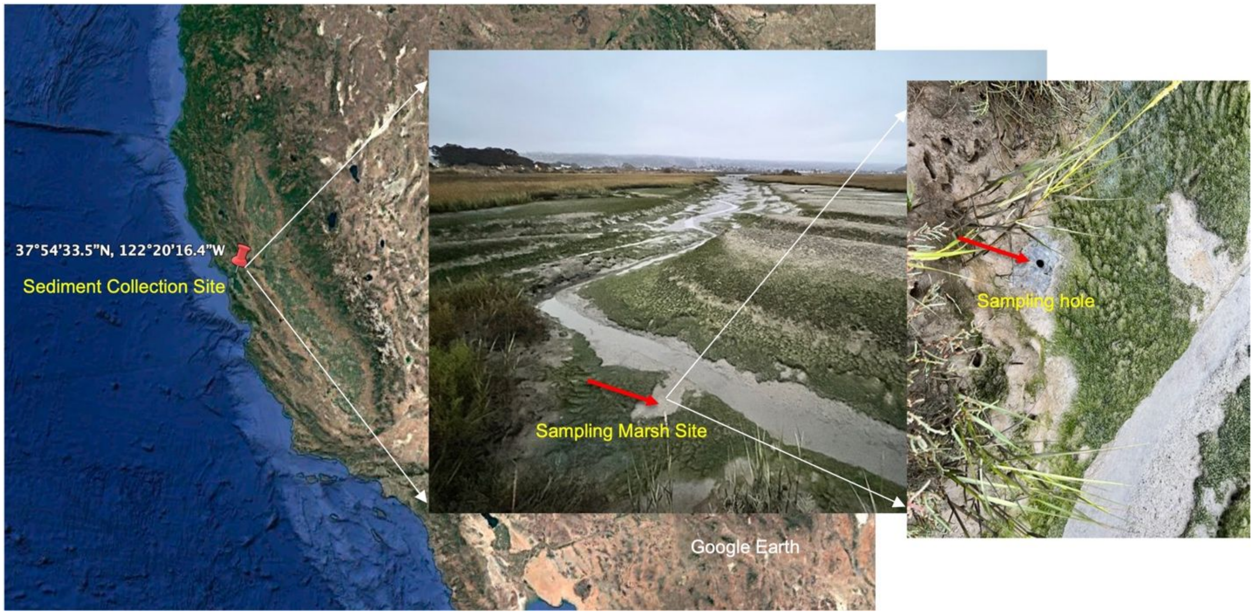

***Figure S1***. *Location and photographs of the coastal sediment collection site (37°54'33.5"N, 122°20'16.4"W) in Richmond, CA.*

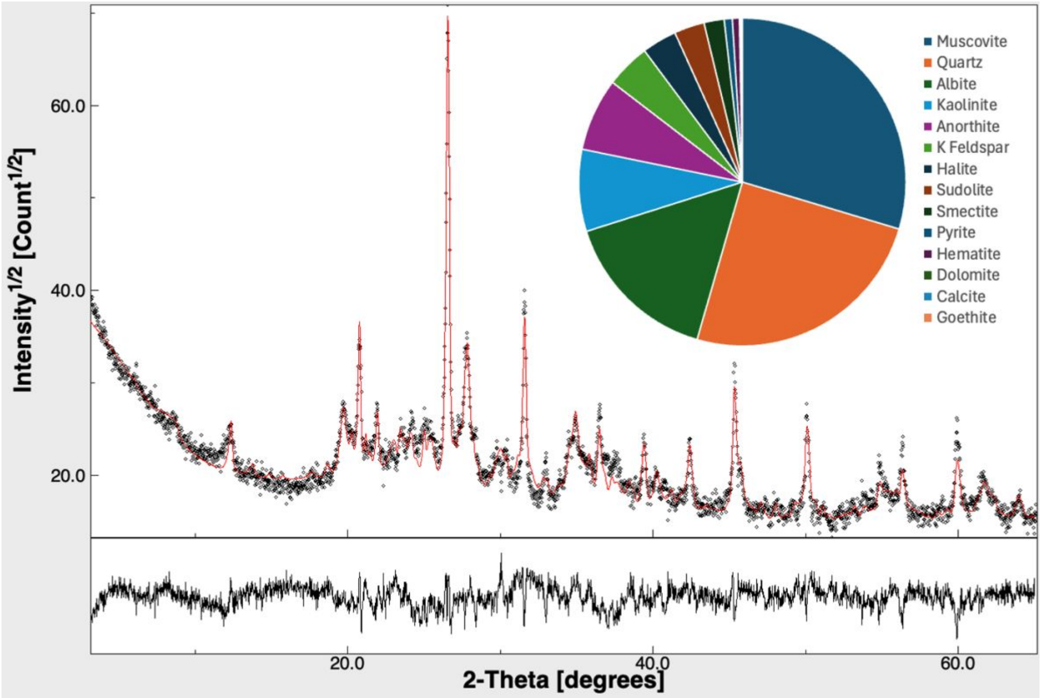


***Figure S2***. *Mineral phase abundance in anaerobic sediment obtained by Reitveld refinement of powder X-ray diffraction data using the MAUD software.*

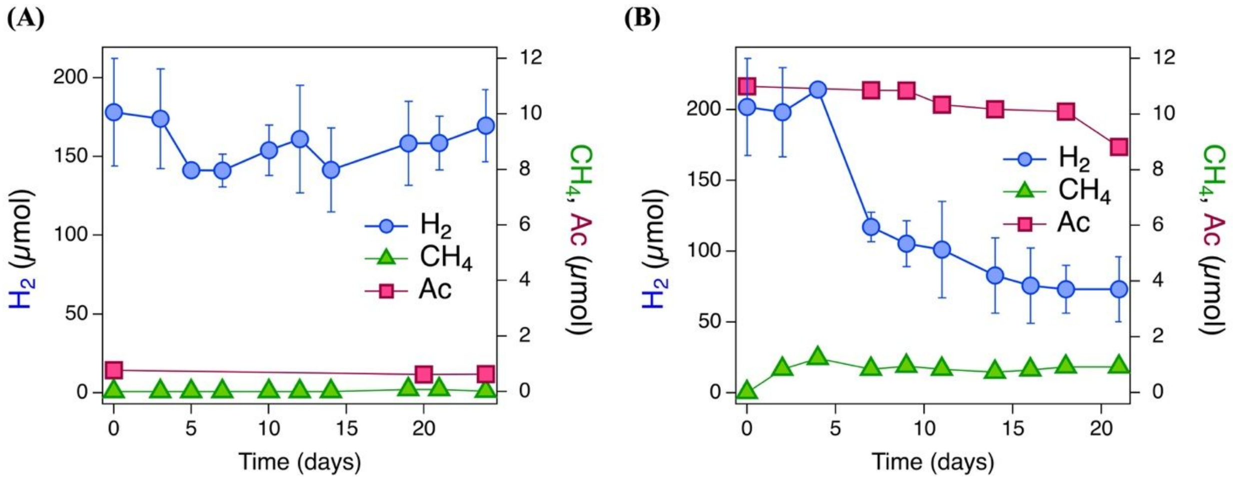


***Figure S3***. *Time dependence of hydrogen ($H_2$, blue circles), methane ($CH_4$, green triangles) and acetate (Ac, pink squares) concentrations in* ***(A)*** *Control experiment of enriched sediment incubated only with hydrogen, and* ***(B)*** *Control experiment of enriched sediment incubated with hydrogen and 10 mM sodium bicarbonate.*

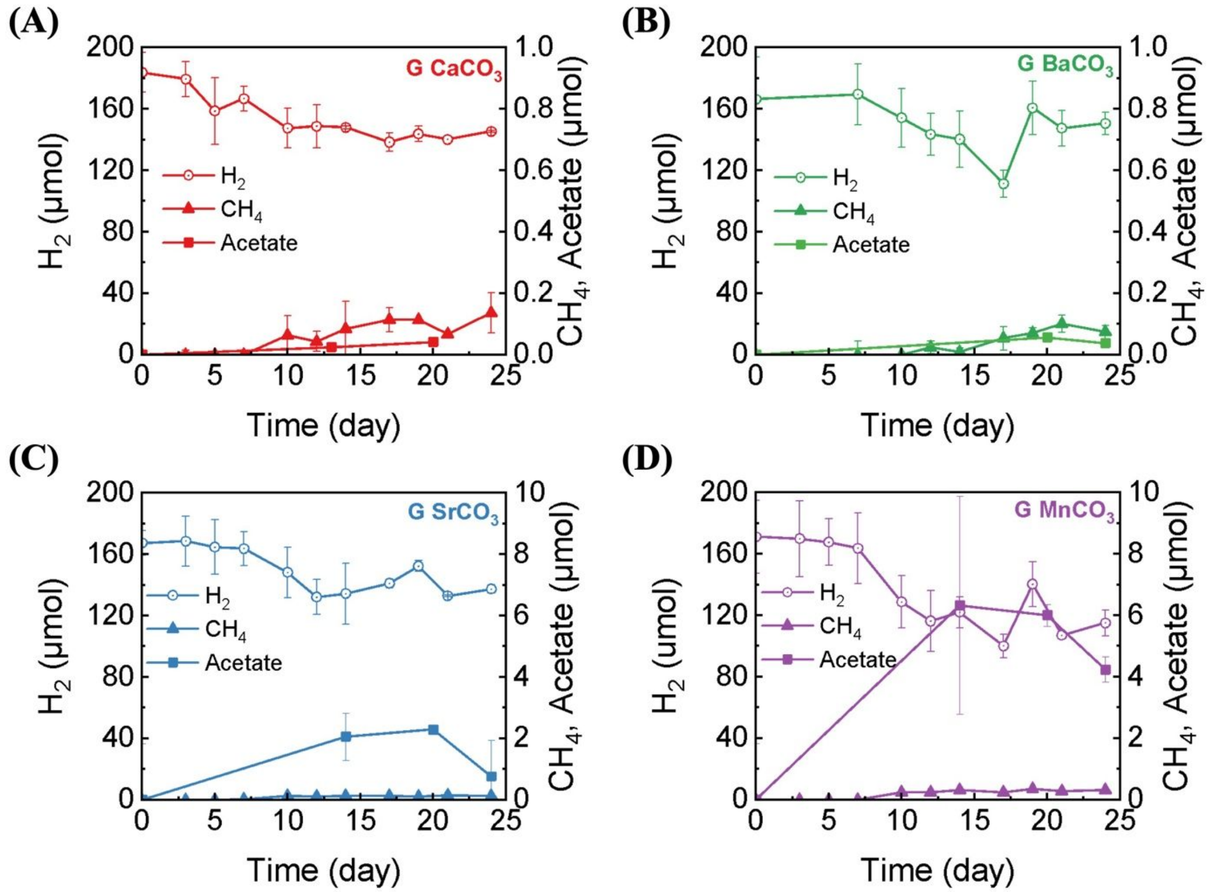


***Figure S4***. *Time dependence of the generation of gas-phase hydrogen and methane and aqueous acetate during the incubation of enriched sediment with 10% hydrogen and 20g/L carbonate minerals (**A**) $CaCO_3$, (**B**) $BaCO_3$, (**C**) $SrCO_3$ and (**D**) $MnCO_3$.*

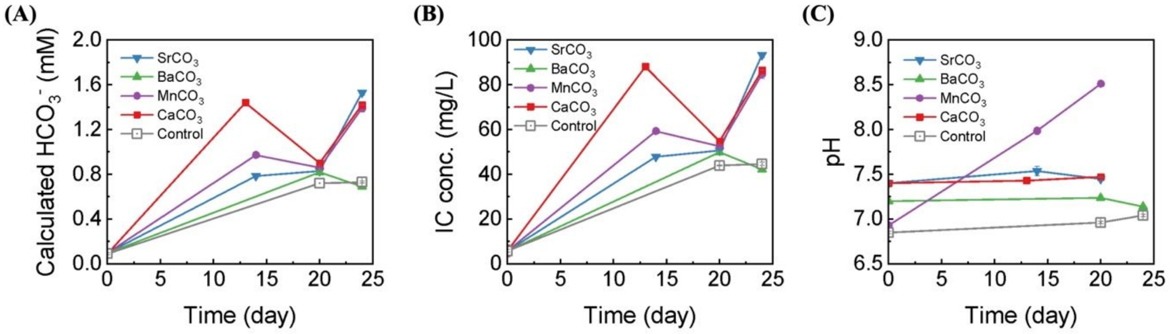


***Figure S5***. *Time dependence of (**A**) calculated concentrations of bicarbonate ($HCO_3^-$), (**B**) measured concentrations of inorganic carbon (IC), and (**C**) measured pH during the incubation of enriched sediment with hydrogen and the carbonate minerals. The concentrations of $HCO_3^-$ were calculated from IC and pH.*

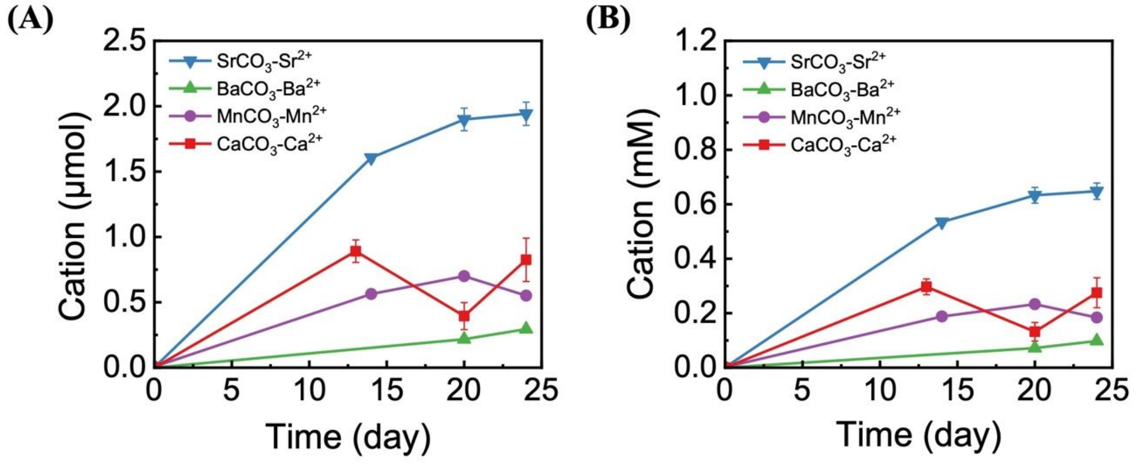


***Figure S6****. The dynamics of (A) carbonate mineral derived cation in micromoles and (B) cation concentrations in incubations with carbonate minerals.*

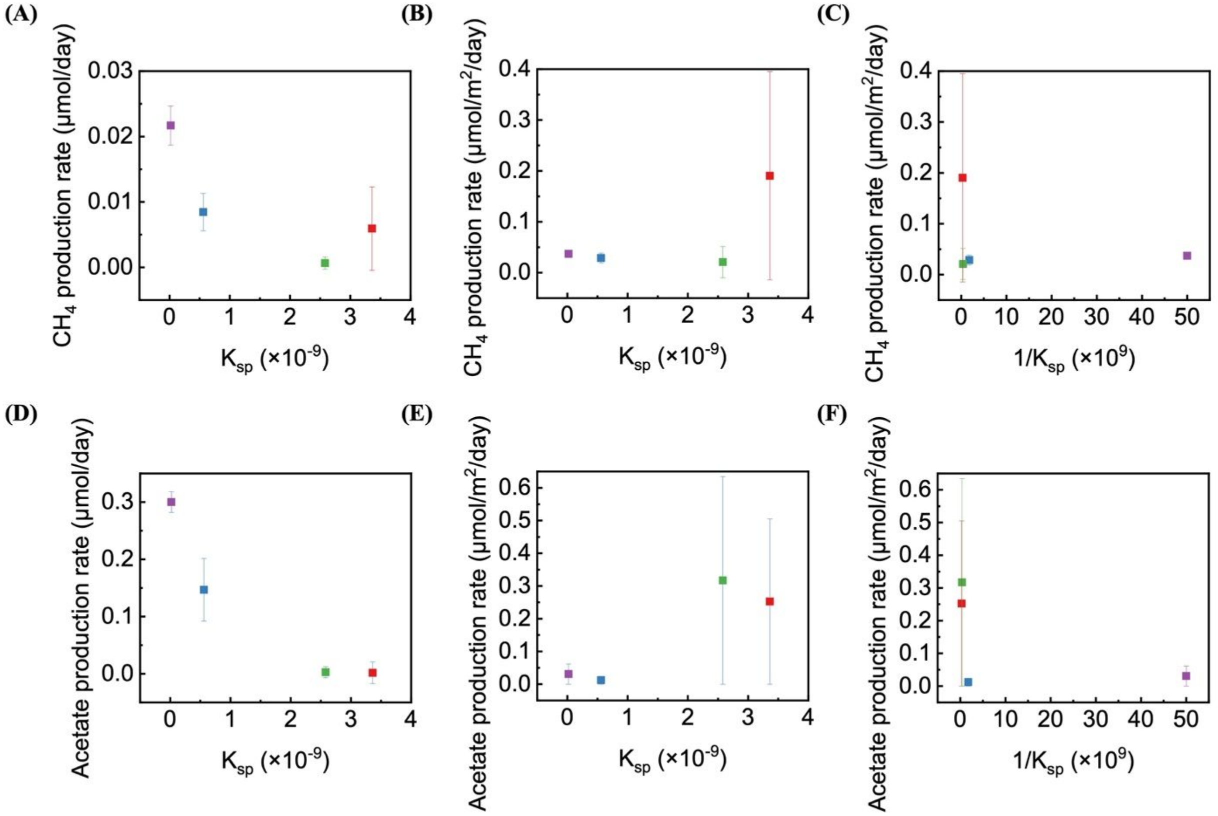


***Figure S7***. *Initial rates and normalized initial rates of hydrogenotrophic (**A**), (**B**), and (**C**) methanogenesis and (**D**), (**E**), and (**F**) acetogenesis correlated to the carbonate mineral solubility (25 ℃, **Table S2**) and the inverse of solubility.*

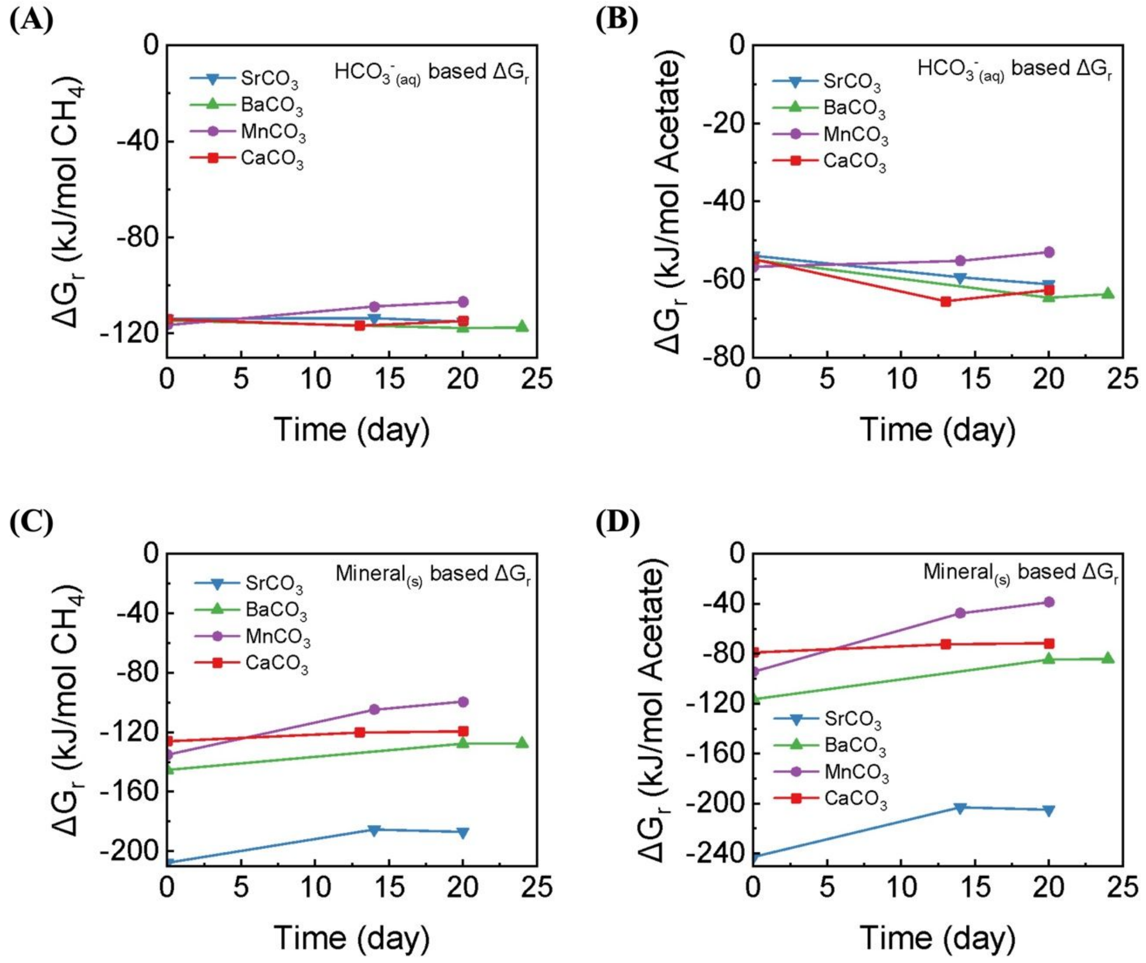


***Figure S8***. *The dynamics of free energy changes, ΔG$_r$, of hydrogenotrophic methanogenesis and acetogenesis calculated based on (**A**) consumption of bicarbonate ions for methane production and (**B**) acetate production (**Table S5**), and (**C**) consumption of carbonate minerals for methane production and (**D**) acetate production (**Tables S6-7**).*

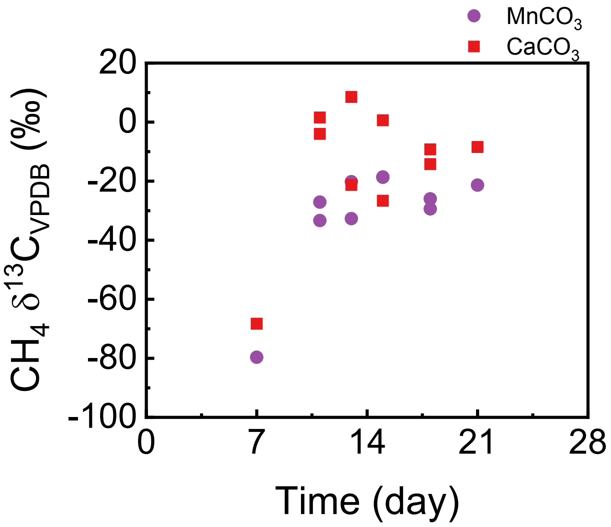


***Figure S9***. *Measured time-dependence of carbon isotope compositions of $CH_4$ ($\delta^{13}C_{VPDB}$ values) during hydrogenotrophic methanogenesis by incubating enriched sediments with carbonate minerals $CaCO_3$ (red) and $MnCO_3$ (purple).*

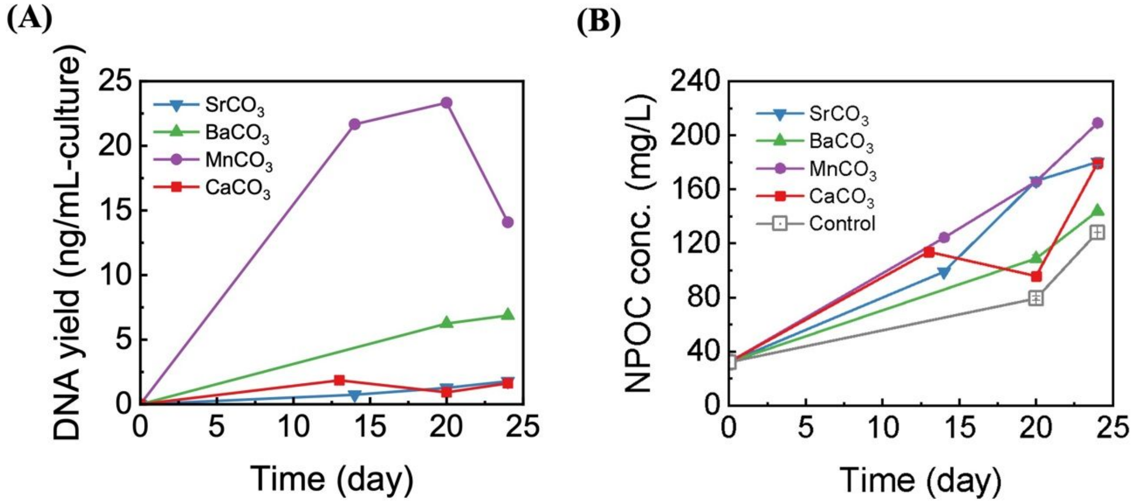


***Figure S10***. *Time dependence of (**A**) the concentration of extracted DNA and (**B**) the concentration of total organic carbon, measured as non-purgeable organic carbon (NPOC), during the incubation of enriched sediment with hydrogen and the carbonate minerals* $SrCO_3$ *(blue),* $BaCO_3$ *(green),* $MnCO_3$ *(purple), and* $CaCO_3$ *(red), and for control experiments (white).*

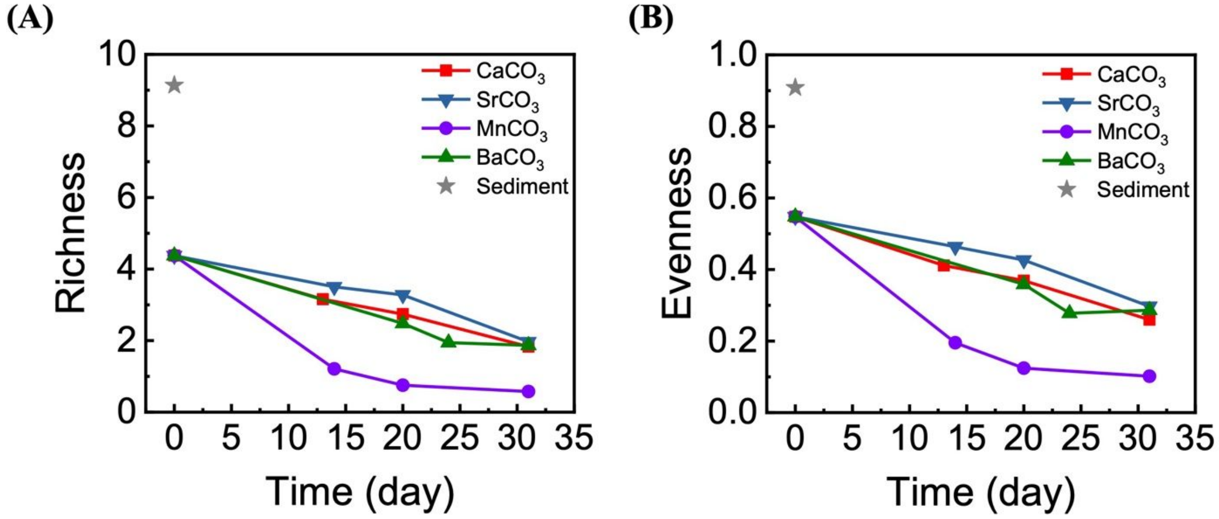


***Figure S11***. *Time dependence of microbial composition (**A**) richness, and (**B**) evenness in raw sediment (grey star), hydrogen-enriched sediment prior to carbonate mineral addition, and during the incubation of enriched sediment with hydrogen and the carbonate minerals* $CaCO_3$ *(red),* $SrCO_3$ *(blue),* $BaCO_3$ *(green), and* $MnCO_3$ *(purple).*

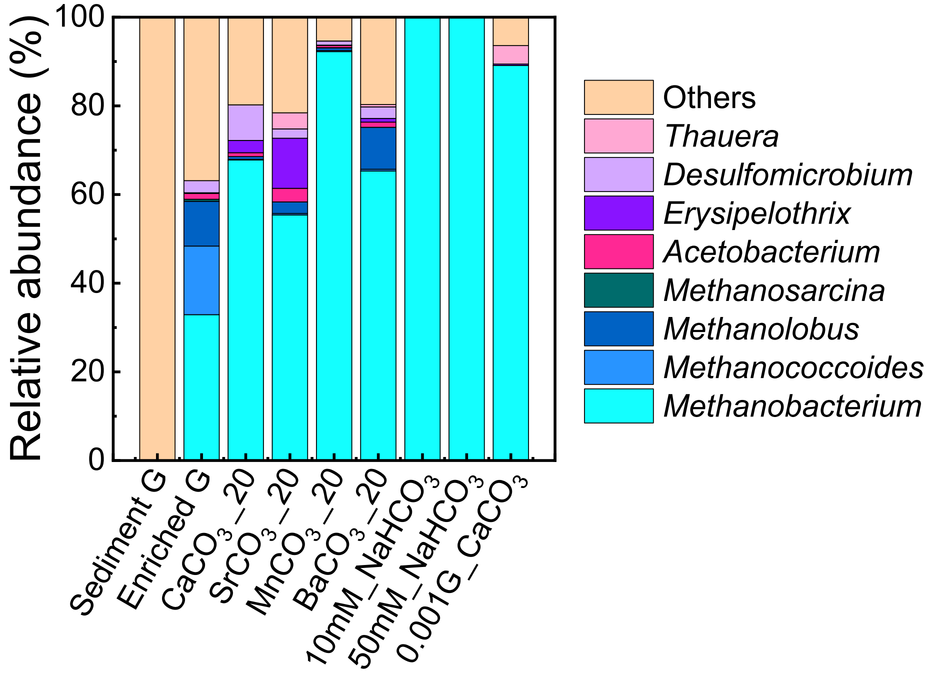


***Figure S12***. *The relative abundance of all identified microorganisms at the genus level in the initial sediment sample G, hydrogen-enriched sediment prior to carbonate mineral addition, and after 20-day incubation of enriched sediment with hydrogen and the carbonate minerals* $CaCO_3$, $BaCO_3$, $SrCO_3$, *or* $MnCO_3$ *or sodium bicarbonate of two concentrations.*

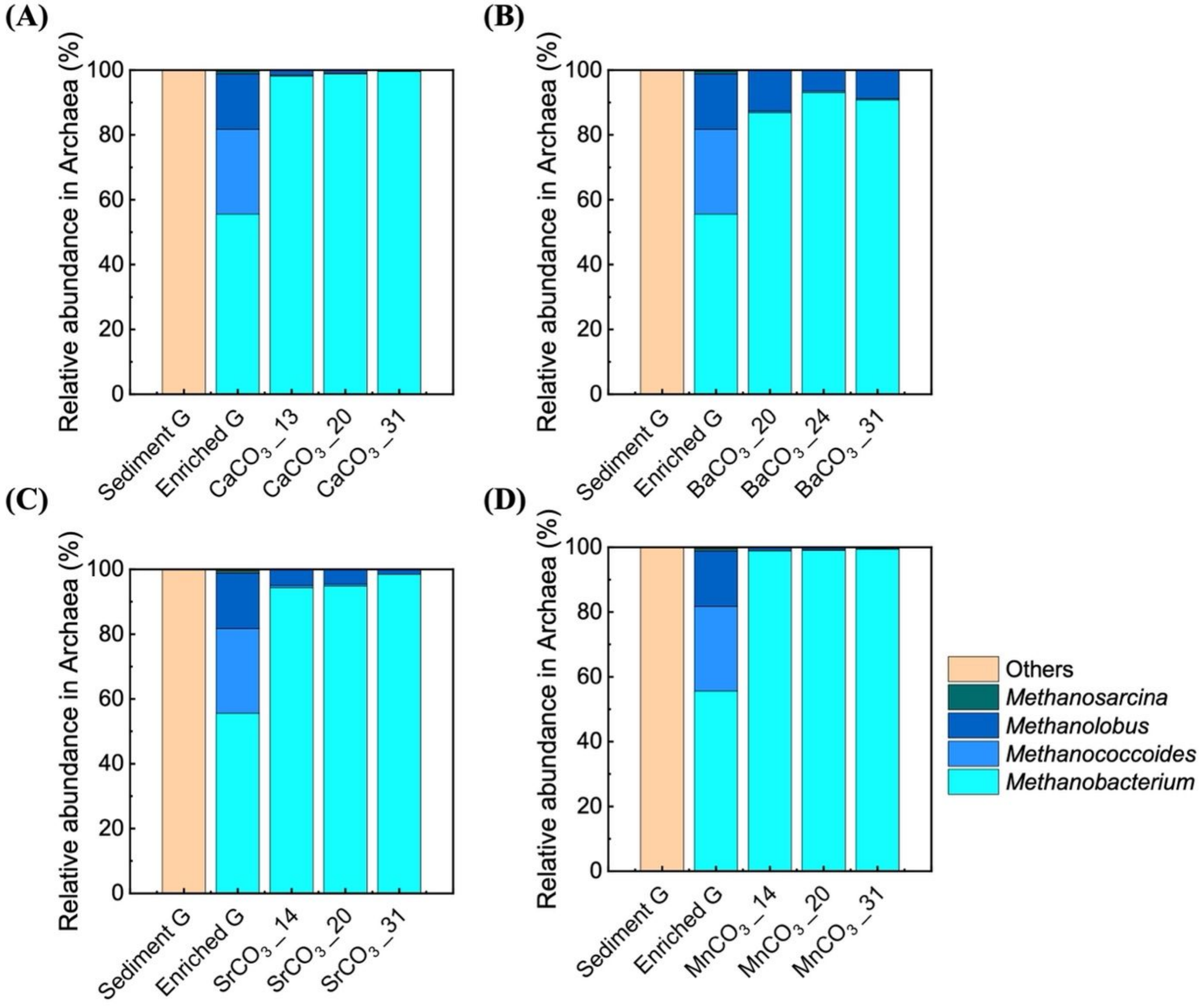


***Figure S13***. *Time-dependence of the relative abundance of microorganisms in the domain of Archaea at a genus level in raw sediment, hydrogen-enriched sediment prior to carbonate mineral addition, and during the incubation of enriched sediment with hydrogen and the carbonate minerals (**A**) $CaCO_3$, (**B**) $BaCO_3$, (**C**) $SrCO_3$, and (**D**) $MnCO_3$. The sampling time after incubation started, in days, is given on the y-axis labels.*

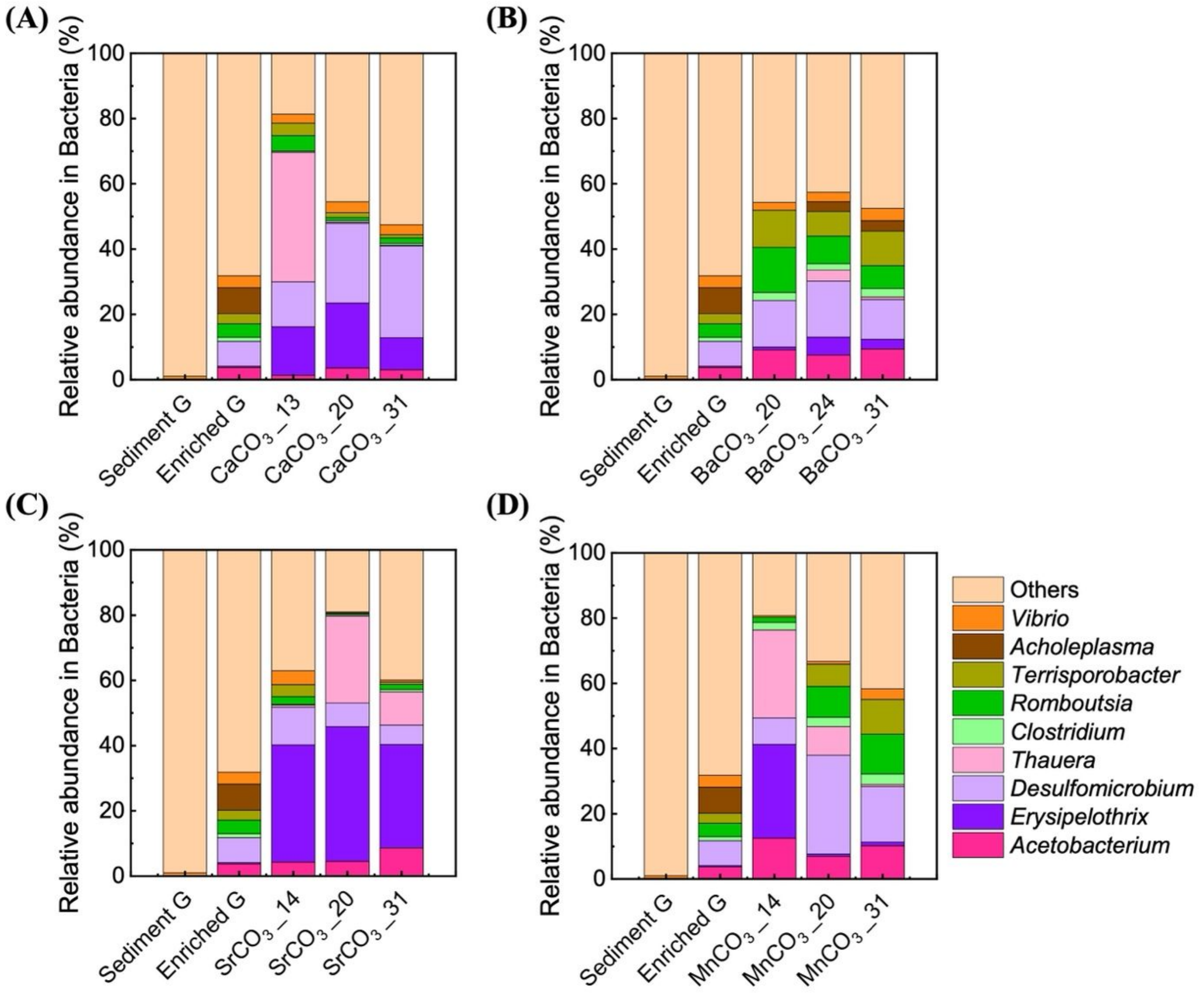


***Figure S14**. Time-dependence of the relative abundance of microorganisms in the domain of Bacteria at a genus level in raw sediment, hydrogen-enriched sediment prior to carbonate mineral addition, and during the incubation of enriched sediment with hydrogen and the carbonate minerals (**A**) $CaCO_3$, (**B**) $BaCO_3$, (**C**) $SrCO_3$, and (**D**) $MnCO_3$. The sampling time after incubation started, in days, is given on the y-axis labels.*

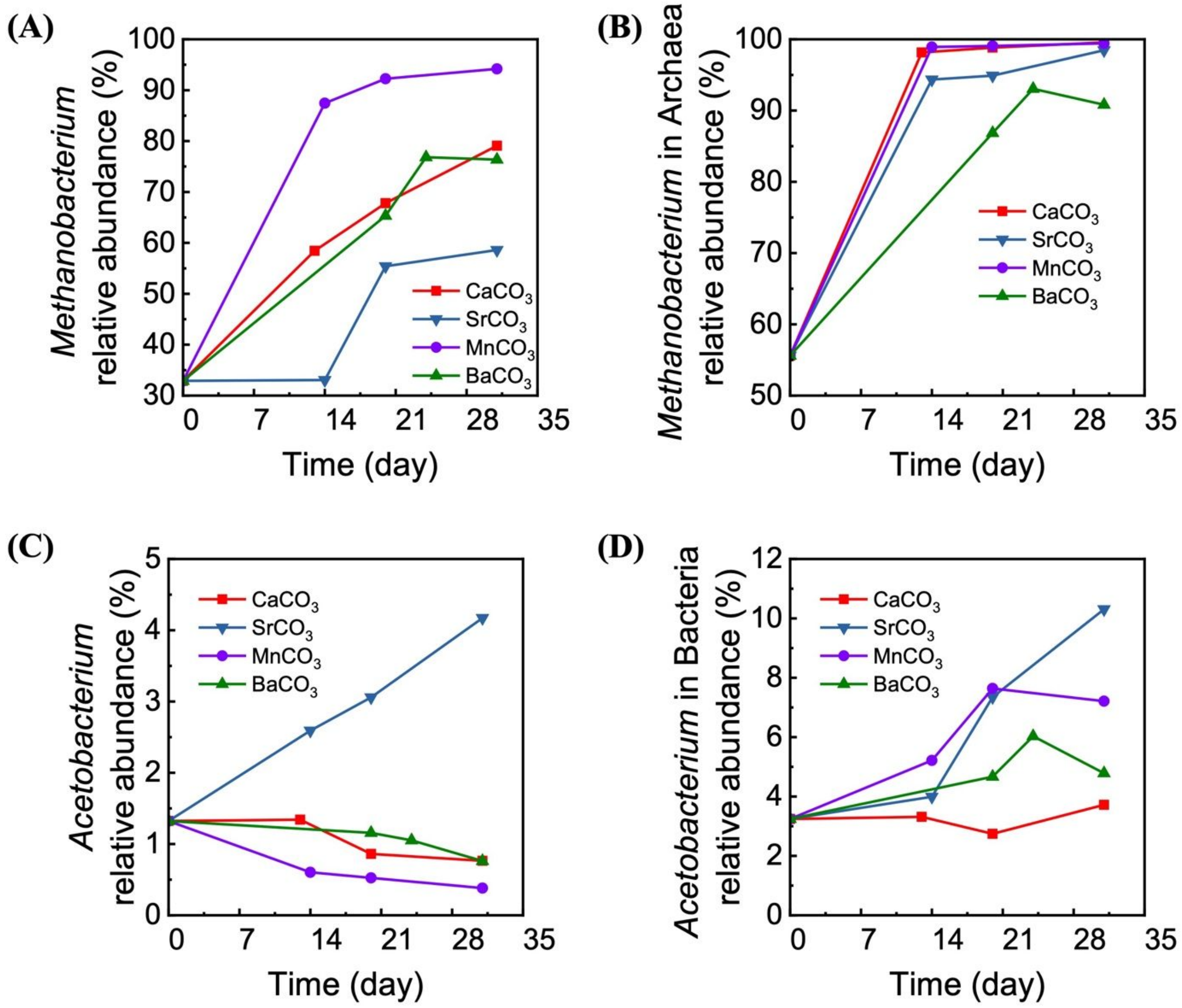


***Figure S15**. **Top**: The relative abundance of Methanobacterium at a genus level (**A**) relative to all identified microbial community members and (**B**) relative to members in the domain of Archaea at a genus level. **Bottom**: The relative abundance of Acetobacterium at a genus level (**C**) relative to all identified microbial community members and (**D**) relative to members in the domain of Bacteria at a genus level.*

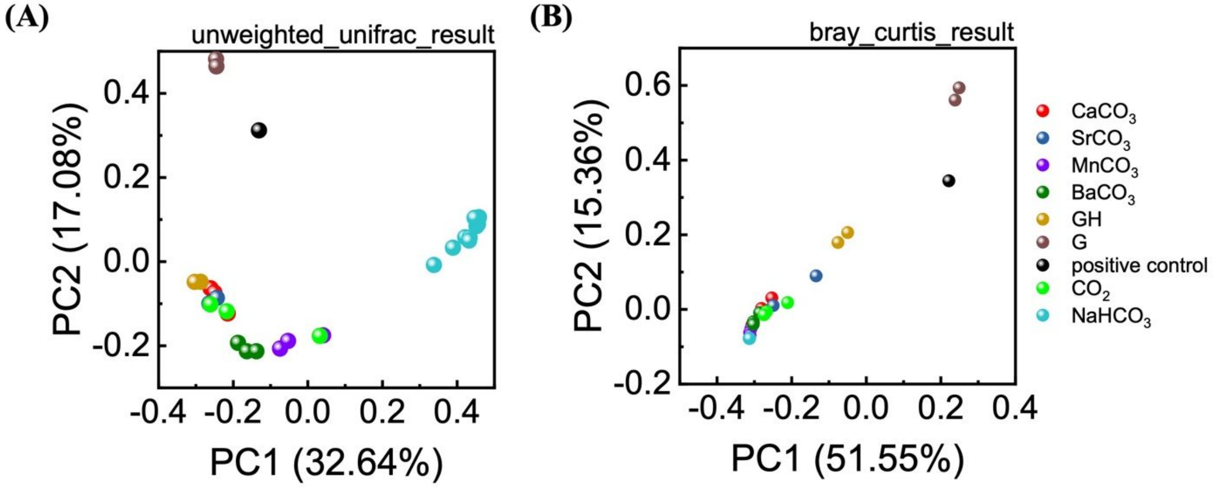


***Figure S16***. *Similarity and dissimilarity of samples based on (**A**) unweighted qualitative phylogenetic beta diversity, and (**B**) quantitative beta diversity measures weighted by taxon abundance (Bray-Curtis). Symbol notations: G denotes raw sediment; GH indicates hydrogen enriched sediment; and* $CaCO_3$, $BaCO_3$, $SrCO_3$, *and* $MnCO_3$ *represent enriched sediment incubations with carbonates of* $CaCO_3$, $BaCO_3$, $SrCO_3$, *and* $MnCO_3$ *respectively;* $CO_2$ *and* $NaHCO_3$ *denote enriched sediment incubations with carbon dioxide and sodium bicarbonate; and positive control denotes bacterial DNA control.*

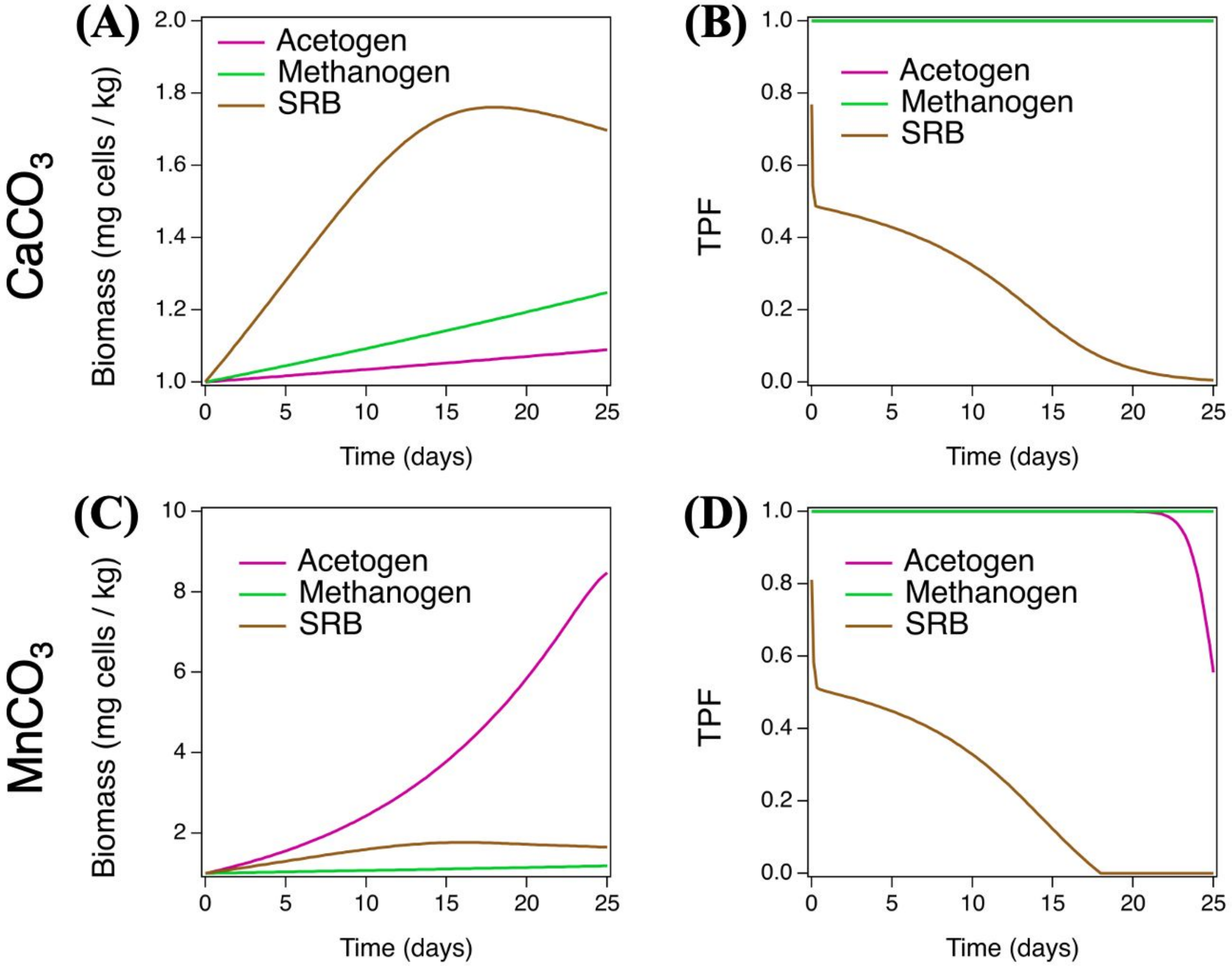


***Figure S17***. *Additional biogeochemical modeling results showing dynamics of (**A**) the biomass growth of acetogen, methanogen, and sulfate reduction bacteria (SRB) and (**B**) the thermodynamic potential factor (TPF) of acetogenesis, methanogenesis, and sulfate reduction for hydrogenotrophy with calcite ($CaCO_3$) and (**C**) and (**D**) that for rhodochrosite ($MnCO_3$).*

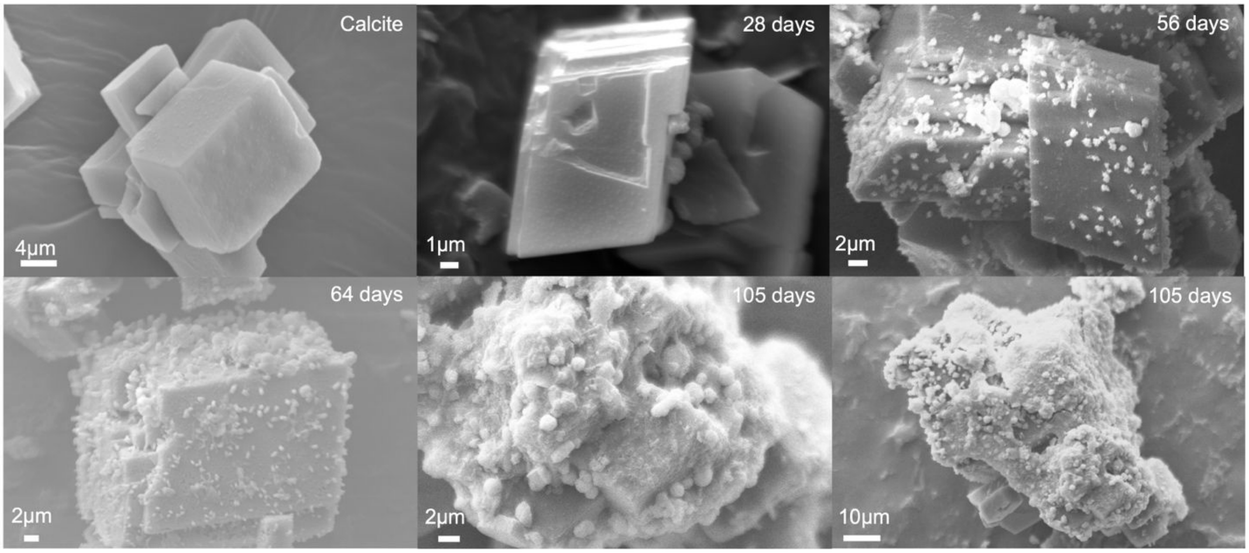


***Figure S18***. *SEM images showing biofilm development on calcite surfaces over 105 days of incubation of diluted enriched sediment with calcite in inorganic medium. This incubation was performed with 10 times smaller quantity of sediment compared to the principal study series.*

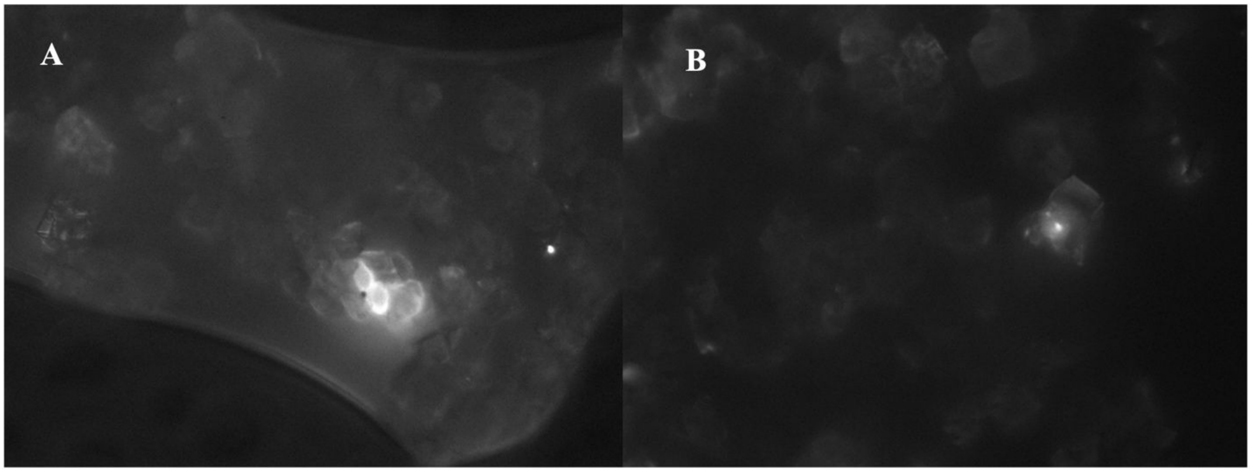


***Figure S19***. *Images (A) and (B) of biofilm with $F_{420}$ autofluorescence on calcite surfaces by fluorescent imaging.*

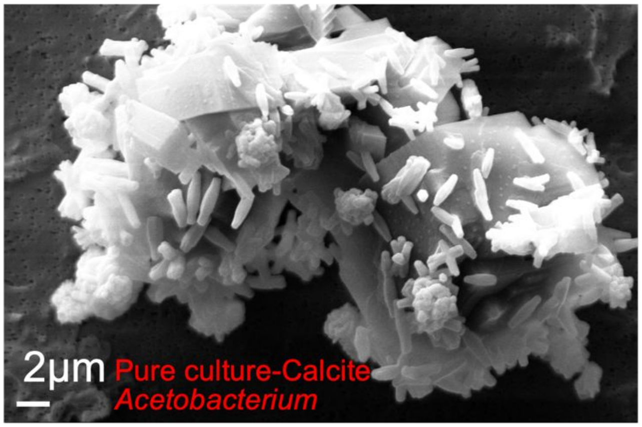


***Figure S20***. *SEM image of acetogen-calcite association from Acetobacterium wieringae pure culture incubation with calcite.*

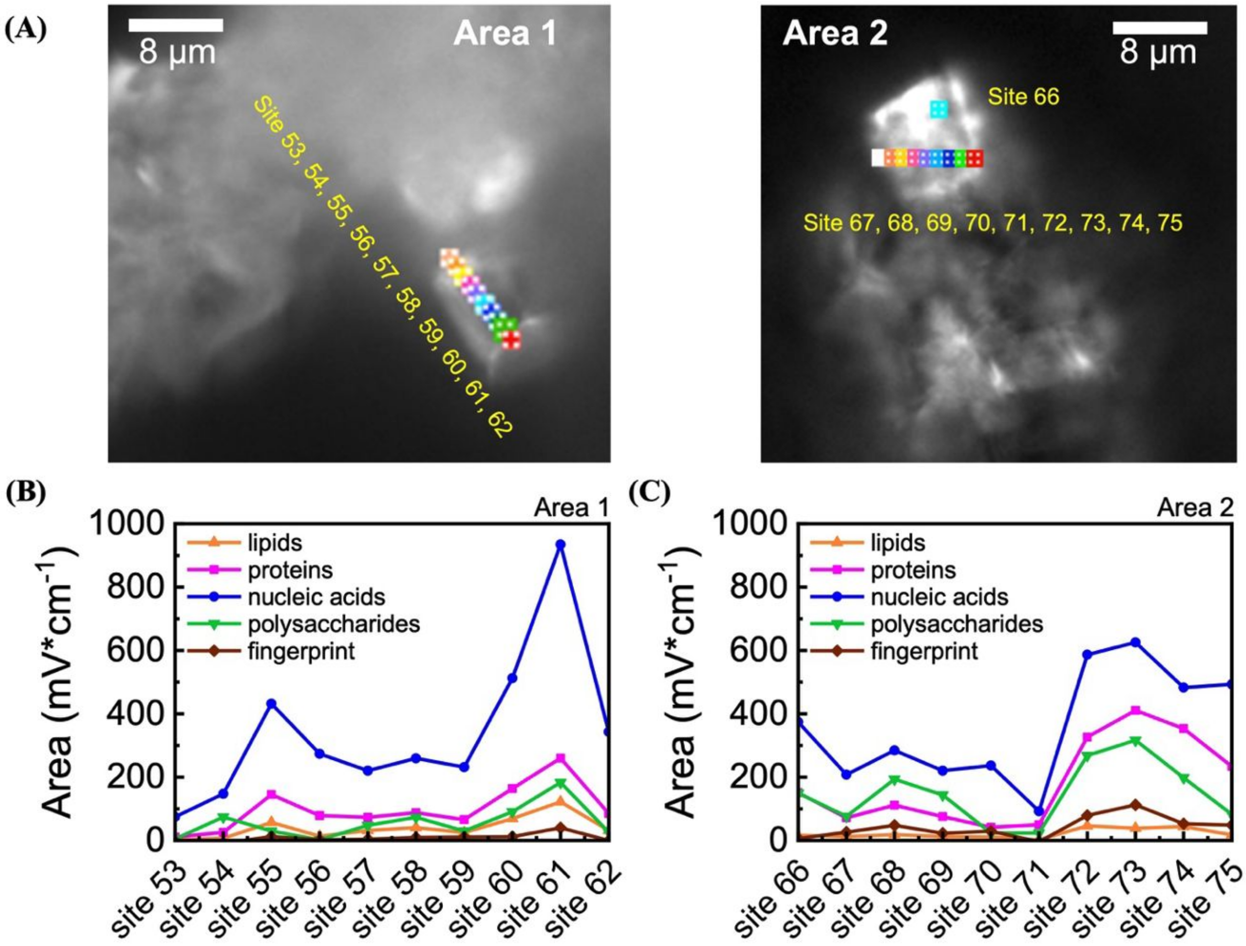


***Figure S21****. Optical photothermal infrared (O-PTIR) spectroscopy results of biofilm on calcite surfaces after incubation of diluted enriched sediment with calcite in inorganic medium. (**A**) Visible light microscopy image of scan Areas 1 and 2 showing the sites where O-PTIR spectra were acquired. (**B**) Relative signals from organic molecule classes for each site in Area 1. (**C**) Relative signals from organic molecule classes for each site in Area 2.*

## References

(1) Bethke, C. M.; Farrell, B.; Sharifi, M. *GWB Reaction Modeling Guide*; 2022.
(2) Dale, A. W.; Regnier, P.; Van Cappellen, P. Bioenergetic controls on anaerobic oxidation of methane (AOM) in coastal marine sediments: a theoretical analysis. *American Journal of Science* **2006**, *306* (4), 246-294.
(3) Bethke, C. M.; Sanford, R. A.; Kirk, M. F.; Jin, Q.; Flynn, T. M. The thermodynamic ladder in geomicrobiology. *American Journal of Science* **2011**, *311* (3), 183.
(4) Jin, Q.; Roden, E. E. Microbial physiology-based model of ethanol metabolism in subsurface sediments. *Journal of contaminant hydrology* **2011**, *125* (1-4), 1-12.
(5) Laura, M.; Jo, P. No acetogen is equal: Strongly different H2 thresholds reflect diverse bioenergetics in acetogenic bacteria. *Environmental Microbiology* **2023**, *25* (10), 2032-2040.
(6) Bahram, M.; Anslan, S.; Hildebrand, F.; Bork, P.; Tedersoo, L. Newly designed 16S rRNA metabarcoding primers amplify diverse and novel archaeal taxa from the environment. *Environmental microbiology reports* **2019**, *11* (4), 487-494.
(7) Takahashi, S.; Tomita, J.; Nishioka, K.; Hisada, T.; Nishijima, M. Development of a prokaryotic universal primer for simultaneous analysis of Bacteria and Archaea using next-generation sequencing. *PloS one* **2014**, *9* (8), e105592.
(8) Bolyen, E.; Rideout, J. R.; Dillon, M. R.; Bokulich, N. A.; Abnet, C. C.; Al-Ghalith, G. A.; Alexander, H.; Alm, E. J.; Arumugam, M.; Asnicar, F. Reproducible, interactive, scalable and extensible microbiome data science using QIIME 2. *Nature biotechnology* **2019**, *37* (8), 852.
(9) Andrews, S. FastQC: a quality control analysis tool for high throughput sequencing data. *Github* **2010**.
(10) Ewels, P.; Magnusson, M.; Lundin, S.; Käller, M. MultiQC: summarize analysis results for multiple tools and samples in a single report. *Bioinformatics* **2016**, *32* (19), 3047-3048.
(11) Callahan, B. J.; McMurdie, P. J.; Rosen, M. J.; Han, A. W.; Johnson, A. J. A.; Holmes, S. P. DADA2: High-resolution sample inference from Illumina amplicon data. *Nature methods* **2016**, *13* (7), 581-583.
(12) Bokulich, N. A.; Kaehler, B. D.; Rideout, J. R.; Dillon, M.; Bolyen, E.; Knight, R.; Huttley, G. A.; Gregory Caporaso, J. Optimizing taxonomic classification of marker-gene amplicon sequences with QIIME 2's q2-feature-classifier plugin. *Microbiome* **2018**, *6*, 1-17.
(13) Thauer, R. K.; Jungermann, K.; Decker, K. Energy conservation in chemotrophic anaerobic bacteria. *Bacteriological reviews* **1977**, *41* (1), 100-180.
(14) Heimann, A.; Jakobsen, R.; Blodau, C. Energetic constraints on H2-dependent terminal electron accepting processes in anoxic environments: a review of observations and model approaches. *Environmental Science & Technology* **2010**, *44* (1), 24-33.
(15) Lovley, D. R.; Goodwin, S. Hydrogen concentrations as an indicator of the predominant terminal electron-accepting reactions in aquatic sediments. *Geochimica et cosmochimica acta* **1988**, *52* (12), 2993-3003.
(16) Marsh, T. L.; McInerney, M. J. Relationship of hydrogen bioavailability to chromate reduction in aquifer sediments. *Applied and Environmental Microbiology* **2001**, *67* (4), 1517-1521.

(17) Cord-Ruwisch, R.; Seitz, H.-J.; Conrad, R. The capacity of hydrogenotrophic anaerobic bacteria to compete for traces of hydrogen depends on the redox potential of the terminal electron acceptor. *Archives of Microbiology* **1988**, *149*, 350-357.
(18) Berta, M.; Dethlefsen, F.; Ebert, M.; Schäfer, D.; Dahmke, A. Geochemical effects of millimolar hydrogen concentrations in groundwater: an experimental study in the context of subsurface hydrogen storage. *Environmental science & technology* **2018**, *52* (8), 4937-4949.
(19) Karadagli, F.; Rittmann, B. E. Thermodynamic and kinetic analysis of the H 2 threshold for Methanobacterium bryantii MoH. *Biodegradation* **2007**, *18*, 439-452.
(20) Wormald, R. M.; Rout, S. P.; Mayes, W.; Gomes, H.; Humphreys, P. N. Hydrogenotrophic Methanogenesis Under Alkaline Conditions. *Front Microbiol* **2020**, *11*, 614227.
(21) Wormald, R. M.; Hopwood, J.; Humphreys, P. N.; Mayes, W.; Gomes, H. I.; Rout, S. P. Methanogenesis from Mineral Carbonates, a Potential Indicator for Life on Mars. *Geosciences* **2022**, *12* (3).
